# Genetic variation in insulin-induced kinase signaling

**DOI:** 10.15252/msb.20156250

**Published:** 2015-07-22

**Authors:** Isabel Xiaorong Wang, Girish Ramrattan, Vivian G Cheung

**Affiliations:** 1 Life Sciences Institute, University of MichiganAnn Arbor, MI, USA; 2 Howard Hughes Medical InstituteChevy Chase, MD, USA; 3 Departments of Pediatrics and Genetics, University of MichiganAnn Arbor, MI, USA

**Keywords:** DNA variants, individual variation, insulin response, signal transduction, type 2 diabetes

## Abstract

Individual differences in sensitivity to insulin contribute to disease susceptibility including diabetes and metabolic syndrome. Cellular responses to insulin are well studied. However, which steps in these response pathways differ across individuals remains largely unknown. Such knowledge is needed to guide more precise therapeutic interventions. Here, we studied insulin response and found extensive individual variation in the activation of key signaling factors, including ERK whose induction differs by more than 20-fold among our subjects. This variation in kinase activity is propagated to differences in downstream gene expression response to insulin. By genetic analysis, we identified *cis*-acting DNA variants that influence signaling response, which in turn affects downstream changes in gene expression and cellular phenotypes, such as protein translation and cell proliferation. These findings show that polymorphic differences in signal transduction contribute to individual variation in insulin response, and suggest kinase modulators as promising therapeutics for diseases characterized by insulin resistance.

## Introduction

Insulin is a key hormone that regulates glucose metabolism; it is essential to support all human cells. Inappropriate insulin sensitivity underlies diseases from diabetes to polycystic ovary syndrome and cancer. Diabetes alone affects nearly 400 million people worldwide and accounts for about 5 million deaths annually (International Diabetes Federation, 2013). Insulin orchestrates a network of signal transducers and gene expression regulators to respond to energy demands and maintain cellular glucose levels. This pathway maintains routine cellular functions, responds to a wide range of energy needs and adapts to changes in glucose levels resulting from food intake.

We differ in our ability to process nutrients. Some people are more and others are less sensitive to glucose and other components in our food. Studies have shown individuals differ extensively in response to glucose load and sensitivity to insulin (Clausen *et al*, 1996; Bouatia-Naji *et al*, 2008). But how these metabolic differences affect one’s well-being and disease susceptibility remains largely unknown. Knowledge of individual variation in insulin response is an important step toward understanding insulin sensitivity. Some gene mutations that influence insulin sensitivity have been identified. For example, mutations in the insulin receptor result in disorders such as the Donohue syndrome (Taylor *et al*, 1981). But these are rare and do not explain more prevalent diseases such as diabetes. Finding the susceptibility genes for common disorders characterized by aberrant insulin response has been difficult. Under the label of insulin resistance is a wide range of abnormalities from inappropriate secretions to decreased insulin sensitivity. Such heterogeneity makes it difficult to identify the genetic basis. Thus, despite several very large-scale genetic studies, the identified genetic variants can only explain about 20% of the disease risks (Drong *et al*, 2012; DIAbetes Genetics Replication And Meta-analysis (DIAGRAM) Consortium *et al*, 2014).

Given the complexity of insulin response, knowledge on which steps along the pathway show the largest individual variability will enable the development of more precise diagnostic tools and therapeutics. More focused genetic studies based on molecular knowledge of insulin response can provide such information. In parallel to genetic studies, other studies have provided molecular and cellular bases of insulin response. Details on how ligand binding triggers insulin receptor to auto-phosphorylate and activate effector proteins such as the insulin receptor substrates (IRS), which then lead to steps that regulate glucose and lipid homeostasis, have been elucidated (Boulton *et al*, 1991; Burgering & Coffer, 1995; Prudente *et al*, 2009). This knowledge can be leveraged to identify the genetic basis of insulin sensitivity. Studies have already shown that dysregulation of phosphoinositide-3 kinase (Hansen *et al*, 1997; Engelman *et al*, 2006) and mitogen-activated protein kinase pathways (Bost *et al*, 2005; Wu *et al*, 2006; Jiao *et al*, 2013) lead to defective insulin response and subsequent insulin resistance. The importance of kinase signaling in diabetes and related disorders is further illustrated by anti-diabetic drugs, such as thiazolidinediones and metformin, that facilitate glucose transport and insulin response through promoting insulin signaling (Zhou *et al*, 2001; Kim *et al*, 2002). Thus, polymorphic differences in kinase activities likely contribute to the development and progression of insulin-related diseases.

In this study, we examined signaling pathways that are activated upon binding of insulin to its receptor, and studied the downstream effects. We found extensive individual variation in the extent of kinase phosphorylation and demonstrated that this propagates to downstream differences in gene expression and cellular response. Among the identified target genes of insulin-induced signaling are diabetes susceptibility genes such as *THADA*, *CENTD2* and *VEGFA*. We confirmed the signaling proteins and target gene relationships by pharmacologic inhibitions and gene silencing. We then mapped DNA variants that influence kinase activations in *cis* and the corresponding downstream response pathways in *trans*. Our results show that individual differences in insulin response begin as early as activation of signal transduction following insulin binding to its receptor and this variation affects gene and cellular responses.

## Results

### Insulin-induced kinase phosphorylation

**Figure 1:**
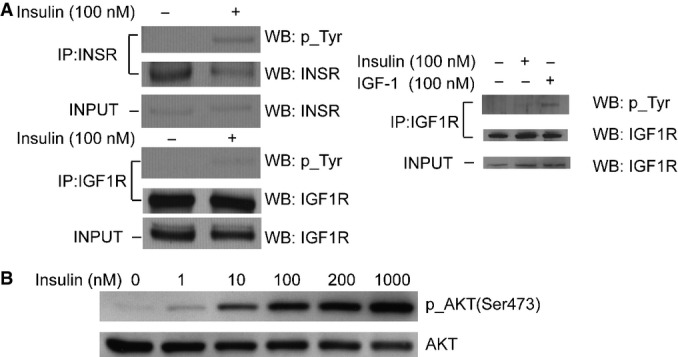
Insulin specifically activates insulin receptor in fibroblasts Insulin treatment induced tyrosine phosphorylation of the insulin receptor (INSR), but not the IGF1 receptor (IGF1R). INSR and IGF1R were pulled down using specific antibodies, and activation of each receptor was assessed by Western blot analysis with anti-phosphotyrosine antibody. The right panel shows IGF1R is activated by IGF-1 as a control experiment. Fibroblasts were treated with IGF-1 or insulin before IGF1R was immunoprecipitated and analyzed for phosphorylation of tyrosine.

**Figure EV1:**
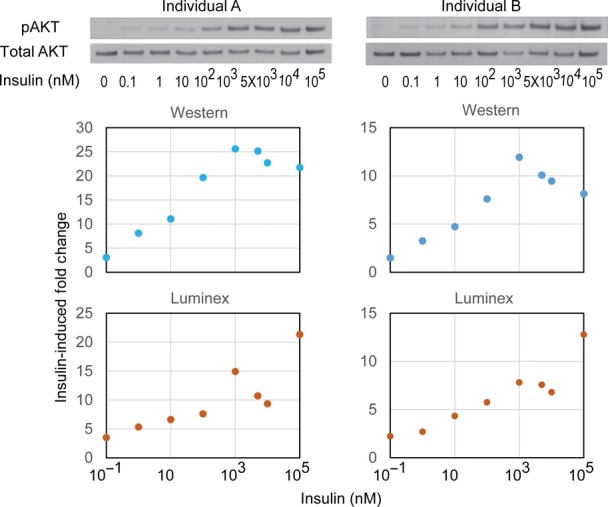
Dose-dependent insulin response in skin fibroblasts Skin fibroblasts from two individuals were treated with a serial dilution of insulin (0.1–10^5^ nM) for 10 min. Whole cell lysates were harvested and analyzed by Western blot and Luminex phosphorylation assay in parallel using antibodies against phospho-Ser473 of AKT or total AKT. Western blot signal was quantified using ImageJ. Fold change of AKT phosphorylation following insulin treatment is shown. Source data are available online for this figure.

To study the response of human cells to insulin and assess individual differences in this response, we treated fibroblasts from 35 age- and gender-matched individuals with insulin and measured kinase activation and changes in gene and protein expression. We used skin fibroblasts since they are easily accessible and are known peripheral targets of insulin action. Skin fibroblasts have been used to study diseases characterized by insulin resistance (Schilling *et al*, 1979; Dunaif *et al*, 1995; Eckardt *et al*, 2007; Porter & Turner, 2009). Human physiological plasma insulin levels are about 0.1–2 nM (Melmed *et al*, 2011); however, we expected a different concentration of insulin would be needed in cell-based experiments. To determine the optimal dose of insulin, we treated cells with serial titration of insulin and chose the concentration in the midpoint of dose–response curve (100 nM), which is the same dose used for cultured fibroblast in other studies (Frittitta *et al*, 1998; Borisov *et al*, 2009). Under our experimental conditions, insulin activates the insulin receptor in fibroblasts but not the insulin-like growth factor 1 receptor (Figs1A and EV1).

Insulin treatment leads to phosphorylation of AKT, a known signaling factor activated by insulin. Cells were treated with a serial titration of insulin for 10 min before they were harvested and analyzed by Western blot. 100-nM insulin treatment was chosen for all following experiments as it is the midpoint of the dynamic range of insulin dose. Source data are available online for this figure.

**Figure 2:**
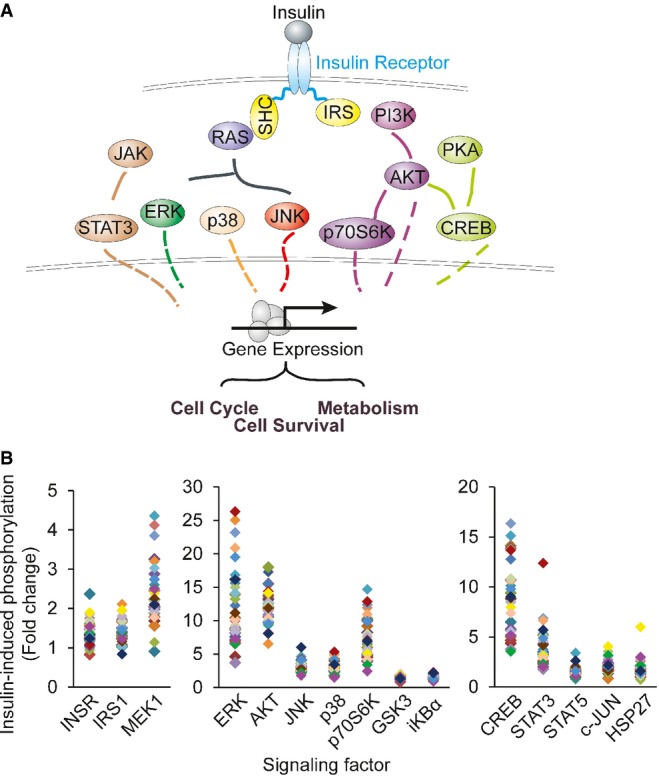
Insulin-induced phosphorylation of multiple signaling factors Schematic diagram of the factors along insulin signaling pathways.

**Figure EV2:**
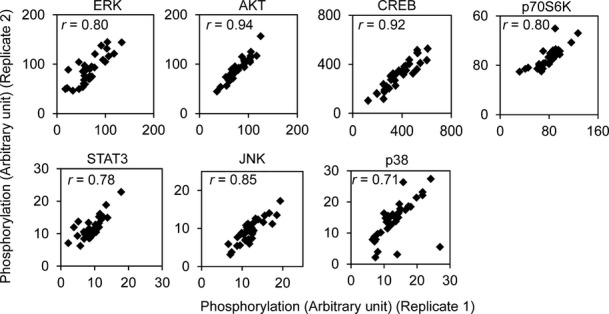
Luminex assay results from biological duplicates are highly similar Fibroblasts from 35 individuals were seeded in duplicate wells, serum-starved and treated with 100 nM insulin for 10 min. Cells were lysed and phosphorylation of each target was measured by Luminex assay.

Binding of insulin to its receptor activates a signaling cascade of factors such as AKT (Figs1B, 2A and EV1). For more quantitative measurements, we used the antibody-based Luminex assay to measure insulin-induced phosphorylation of 15 proteins. The results showed that within 10 min following insulin treatment, several proteins were activated; these include the insulin receptor β subunit (INSRβ) and insulin receptor substrate 1 (IRS-1; Fig2B). Phosphorylation of the 15 signaling proteins increased by 1.4- to over 10-fold. Seven signaling factors (AKT, CREB, ERK, JNK, p38, p70S6K and STAT3) showed an average of 3- or greater fold increase in phosphorylation levels. Results from replicates are highly correlated (*r* > 0.7; Fig EV2). Using Western blot, we validated the induction of phosphorylation measured by Luminex; while results from these two methods are similar, the Luminex assay provides broader dynamic range (FigEV1). Moreover, measurement of the total protein levels showed that even though the levels of phosphorylation increased following insulin, the total protein level did not change (FigEV3). Together, these results identified at least seven signaling proteins activated in response to insulin.

Results from Luminex assays show phosphorylation of the signaling factors 10 min following insulin treatment. Each individual is represented with a different color. Average phosphorylation levels of the biological duplicates from the same individual are shown. Source data are available online for this figure.

**Figure EV3:**
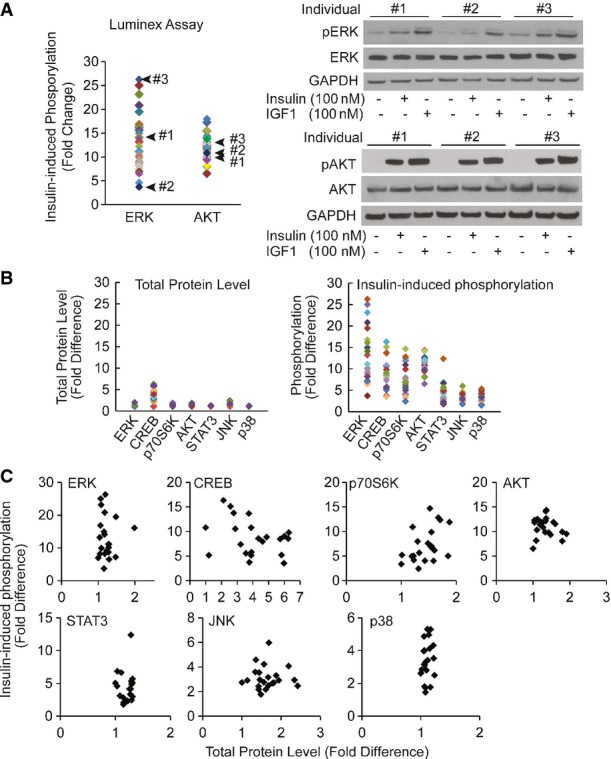
Luminex phosphorylation measurements are reproducible and have broad dynamic range Individual difference in insulin-induced phosphorylation of ERK measured by Luminex assay is validated by Western blot. Skin fibroblasts from the indicated 3 individuals were treated with 100 nM insulin in duplicates. Cells were harvested and assayed using Luminex phosphorylation assay or Western blot using the same antibodies. Cells were treated with 100 nM IGF1 as a positive control. Total protein levels of AKT or ERK did not change before and after insulin treatment.

Compared to differences in phosphorylation levels, total protein levels are not as variable across individuals. Total protein levels or insulin-induced phosphorylation levels of 7 signaling factors shown in Fig5A were measured in 20 individuals by Luminex assay. Average value of biological duplicates is shown.

Fluctuation in total protein levels does not contribute to variation in phosphorylation levels induced by insulin. The same data as in (B) were re-plotted to show there is no correlation between phosphorylation levels and total protein levels. Source data are available online for this figure.

### Insulin-induced changes in gene and protein expression

**Figure 3:**
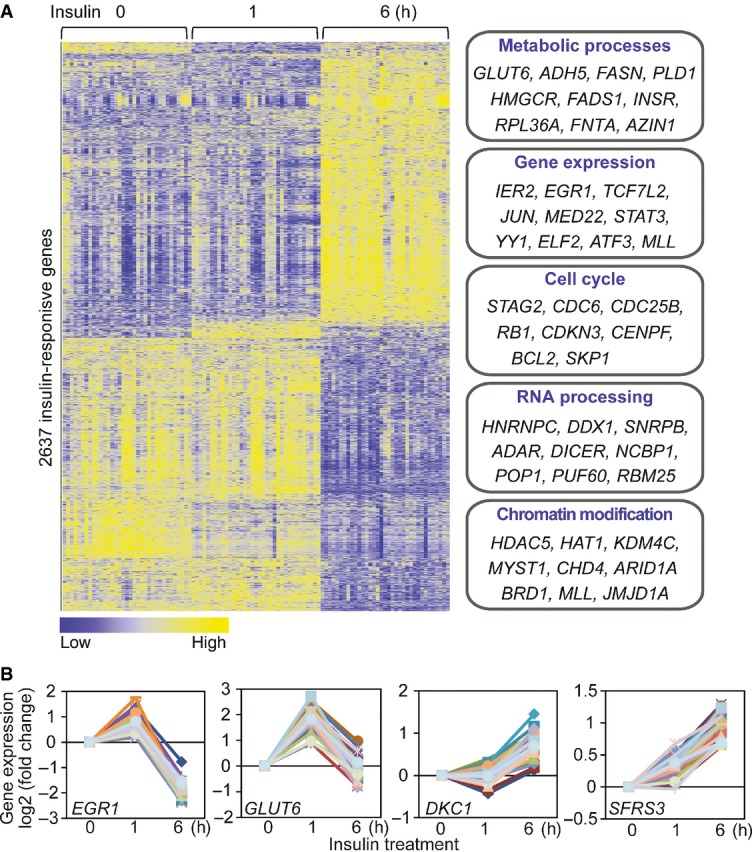
Insulin-induced changes in gene expression Heatmap shows the expression levels of 2,637 insulin-responsive genes (*P* < 10^−6^; ANOVA). Examples of genes that are involved in various biological processes are listed.

To study the downstream effect of kinase activation, we measured gene expression of cells at one and 6 h following insulin treatment. As expected, insulin induced changes in expression of many genes. The expression of 2,637 genes (22% of expressed genes) significantly changed following insulin treatment at one or both time points (*P *< 1 × 10^−6^, ANOVA; Fig3A; Dataset EV1). These include genes that are known to play a role in insulin response, such as immediate early response gene (*IER2*) and early growth response genes (*EGR1*, *EGR2* and *EGR3*), and the glucose transporter type 6 (*GLUT6*) and activating transcription factor 3 (*ATF3*). Gene ontology analysis (Ashburner *et al*, 2000) shows that these “insulin-responsive genes” are significantly enriched for roles in the regulation of cell cycle, RNA processing and metabolism (Benjamini *P* < 10^−10^). By text-mining the literature, we found that many of these insulin-responsive genes have not been implicated in insulin-mediated pathways. A search of PubMed for co-occurrence of the term “insulin” or “diabetes” with each of the 2,637 genes showed that less than half of these genes were mentioned in insulin or diabetes literature; thus, these results identified a large number of insulin-responsive genes. Among those uncovered in this study are *DKC1* that plays a role in maintaining telomere integrity and when mutated leads to dyskeratosis congenita, and *SFRS3*, a serine/arginine-rich splicing factor 3 (Fig3B).

Examples of insulin-responsive genes, including those known to be regulated by insulin (*EGR1*, *GLUT6*), and those that were not previously implicated in insulin response (*DKC1*, *SFRS3*).

**Figure 4:**
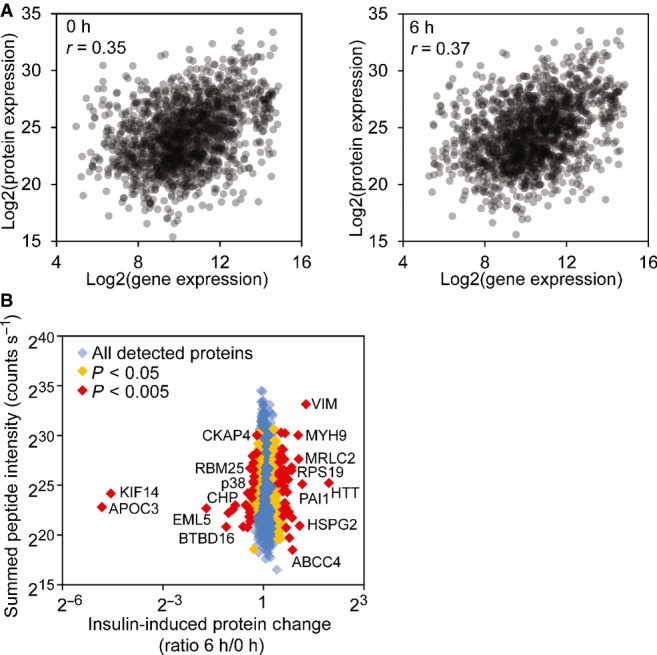
Insulin-induced changes in protein expression Correlations of protein and gene expression levels before and after insulin treatment. The gene expression levels are averages from our 35 subjects.

To extend the study beyond changes in gene expression, we carried out a proteomic analysis of the insulin-treated cells using stable isotope labeling by amino acids in cell culture (SILAC) mass spectrometry (Ong *et al*, 2002). A total of 1,828 proteins (at least two unique peptides per protein) were identified. Among them, the corresponding transcripts for 1,638 proteins were also found in our gene expression study. The gene and protein expression levels of the 1,638 pairs were significantly correlated (*r* = 0.35 and 0.37 at time 0 and 6 h, respectively; *P* < 0.001; Fig4A), consistent with the previous studies of relationships between transcript and protein expressions (Gygi *et al*, 1999; Kislinger *et al*, 2006; De Godoy *et al*, 2008). The expression levels of 158 proteins showed significant changes at 6 h following insulin treatment (*P* < 0.05; Fig4B; Cox & Mann, 2008). These “insulin-responsive proteins” play a role in protein biosynthesis (EIF1, RPS6), RNA processing (RBM25, HNRNPC), lipid biosynthesis (APOC3, FASN) and protein transport (XPO5, TMED3). For instance, it was previously reported that abnormal level of APOC3 led to hypertriglyceridemia in transgenic mice and humans (Carlson & Ballantyne, 1976; Ginsberg *et al*, 1986; Ito *et al*, 1990; Aalto-Setälä *et al*, 1992; Pollin *et al*, 2008). *APOC3* is transcriptionally repressed by insulin, a critical step of its regulation (Chen *et al*, 1994; Li *et al*, 1995). Here we showed that APOC3 protein is down-regulated following insulin treatment, supporting its role in mediating insulin response.

Protein expression changes after insulin treatment quantified by mass spectrometry (SILAC). Examples of proteins with significant changes of expression are annotated (MaxQuant Significance B statistical test). Source data are available online for this figure.

### Individual variation in insulin-induced kinase activation

**Figure 5:**
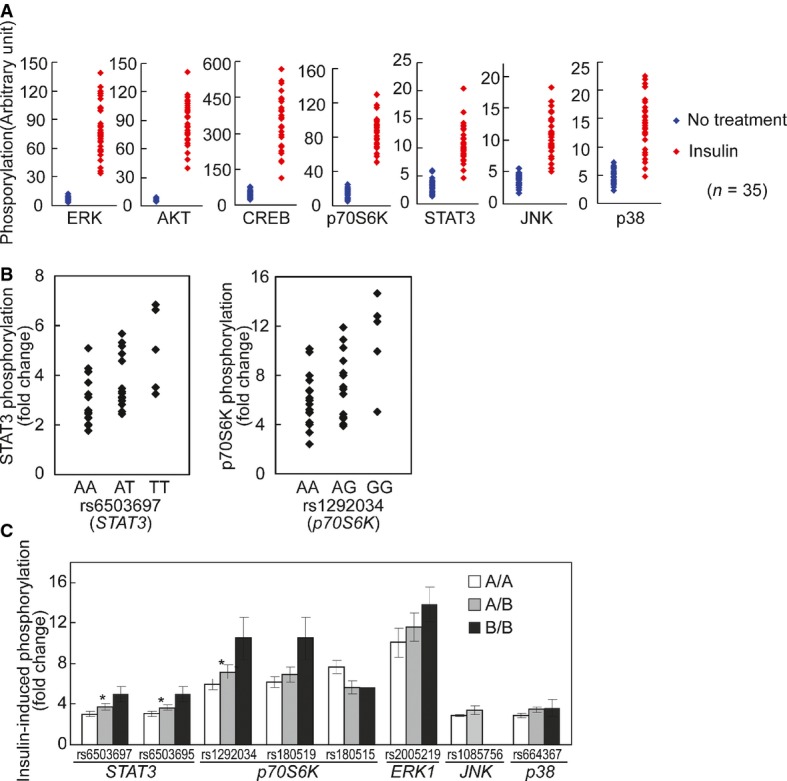
Individual variation in kinase phosphorylation in response to insulin Phosphorylation of signaling factors differs extensively among individuals following insulin treatment compared to those at baseline. The data in Fig2A are re-plotted here to contrast the phosphorylation before and after insulin treatment.

**Figure EV4:**
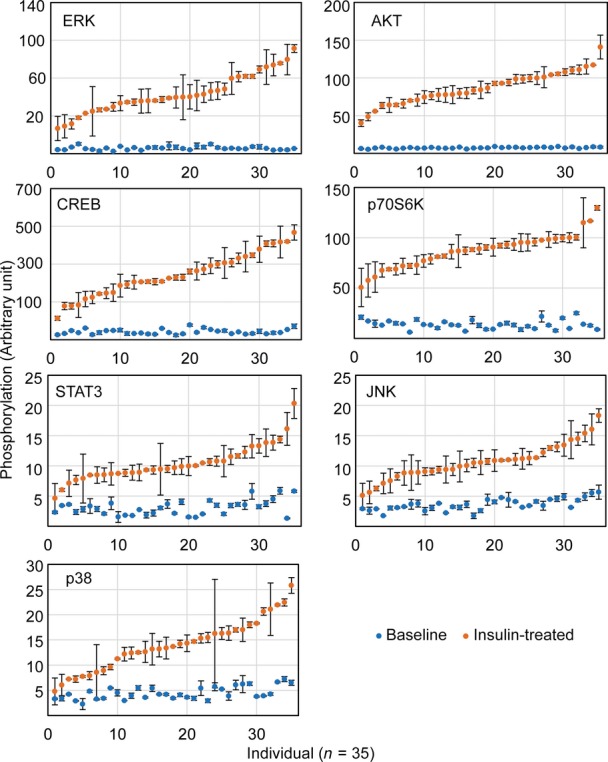
Phosphorylation levels of signaling factors after insulin treatment do not show significant correlations with those at baseline Phosphorylation of each signaling factor at baseline or after insulin treatment from the same individual is shown in the same column. Variation among individuals in insulin-induced phosphorylation is significantly greater than that within replicated assays (*P *< 0.05; ANOVA) for all seven signaling factors. The average phosphorylation level of replicates is shown. Error bars represent SEM of biological replicates.

The above findings were obtained by averaging the results from our subjects. As we studied the data, we noticed individual differences in response to insulin. While all the signaling proteins were activated by phosphorylation in response to insulin, the extent of phosphorylation differs greatly among individuals (Fig2B). When we compare the phosphorylation levels before and after treatment, in un-stimulated cells across all individuals, the kinases were not activated and thus showed little variability (Figs5A and EV4). In contrast, there was extensive individual variation in phosphorylation levels of the signaling factors following insulin treatment. For example, insulin-induced phosphorylation levels of ERK1/2 at Thr185/Tyr187 are highly variable. Among the 35 subjects, the individual with the lowest induction showed a ∼4-fold increase while the person with the highest induction showed a ∼26-fold increase in phosphorylation of ERK. The phosphorylation of Ser473 of AKT increased from 1.7- to 20-fold. All the measurements were made with biological replicates; results between replicates are highly correlated (*r* > 0.7; FigEV2). Variation was significantly higher among individuals than within replicates for all signaling factors (*P* < 0.05, ANOVA; FigEV4). Using Western blot analysis, we confirmed the individual variation in insulin-induced phosphorylation of target protein such as AKT and ERK (FigEV3A). Moreover, the differences in phosphorylation of AKT and ERK are not due to differences in expression levels of total AKT and ERK since those do not vary among individuals, and showed little changes following insulin treatment (FigEV3B and C). These results uncover that upon insulin binding to its receptor, even in the early steps of activation of signal transduction pathway, there are significant individual differences in response to insulin.

*Cis*-regulation of the activation of signaling proteins after insulin treatment. Association analysis shows allelic differences in insulin-induced phosphorylation of STAT3 and p70S6K. The extent of phosphorylation for each signaling protein is plotted by genotypes of 34 individuals (one outlier was removed).

Results from association analysis of additional SNPs. For all SNPs, the order of presentations is AA, AB and BB where A is the common allele and B is the minor allele (* denotes corrected *P*-value < 0.03 in association analysis). Significant allelic associations with *cis*-acting SNPs were found for phosphorylation of STAT3 with rs6503697 (chr17:42349561; Pc = 0.006) and rs6503695 (chr17:42347515; Pc = 0.013) and for phosphorylation of p70S6K with rs1292034 (chr17:59912499; Pc = 0.028).

To understand the basis of the variation in insulin-induced signaling, we carried out genetic analysis. To narrow the search, we tested for allelic associations between DNA sequence variants within the genes that encode the signaling proteins and the extent of insulin-induced phosphorylation. SNPs within the 7 signaling proteins (10 kb upstream of 5′ UTR to 10 kb downstream of 3′ UTR; minor allele frequency > 5%) were selected from dbSNP (Smigielski *et al*, 2000) and genotyped in our subjects. By association analysis, we found that the activation of several signaling proteins was in part *cis*-regulated (Fig5B and C). For example, insulin-induced phosphorylation of STAT3 in cells of TT homozygotes for rs6503697 (chr17:42349561) was 66% higher than that in the AA homozygotes (Pc = 0.006). Similarly, phosphorylation of p70S6K was 75% higher in individuals with GG genotypes at rs1292034 (chr17:59912499) than in the AA individuals (Pc = 0.03). These results show a genetic contribution to individual variability in insulin-activated signaling pathways. Further study is needed in order to identify functional variants that directly affect phosphorylation levels of target proteins.

Previous studies have shown that cells respond to multiple extracellular stimuli through cross talks between different pathways. For instance, the EGF- and insulin-induced signaling pathways share some downstream factors in that insulin treatment enhances ERK phosphorylation stimulated by EGF (Borisov *et al*, 2009). We therefore asked whether variants in EGF receptor gene (*EGFR*) contribute to different levels of insulin-induced phosphorylation. We obtained genotype of 17 SNPs within *EGFR* (minor allele frequency > 5%) and analyzed the association between these variants and phosphorylation of ERK and AKT. Individuals carrying A allele at rs10228436 (chr7:55170575) show higher phosphorylation of AKT than those carrying G allele (nominal *P*-value = 0.02). Similarly, variants at rs4947986 (chr7:55153962) are associated with ERK phosphorylation (nominal *P*-value = 0.03). However, these associations are weak as none of them is significant after correction for multiple testing. Given the cross talks between pathways, it is likely that several regulators in addition to EGF receptor affect ERK and AKT phosphorylation and a larger sample is needed to identify complex genetic interactions.

### Individual variation in insulin-induced gene expression response

**Figure 6:**
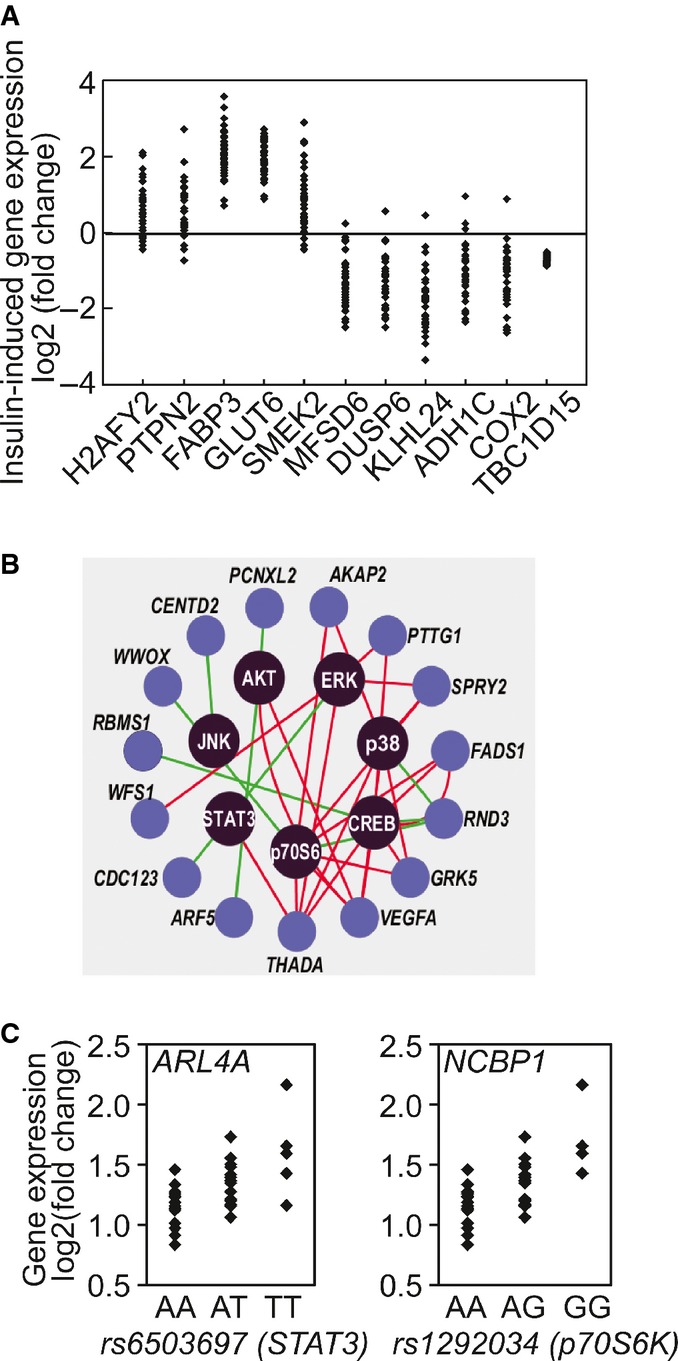
Individual variation in gene expression in response to insulin Example of 10 genes whose gene expression responses following insulin treatment vary among individuals. Changes in the expression of *TBC1D15*, in contrast, are similar among all subjects.

Next, we examined whether insulin-induced gene expression response also varies among individuals. Analysis of gene expression among the 35 individuals showed that like kinase activation, the extent of gene expression changes differs among individuals. A total of 4,455 genes showed at least a 2-fold difference among the individuals with the least and the most induction (or repression) in gene expression following insulin treatment at one or both time points. For example, the expression of *DUSP6* and *COX2* differs by more than 8-fold across the individuals. The ranges of insulin-induced changes in gene expression across individuals for some representative genes are shown in Fig6A.

Insulin-induced changes in expression levels of susceptibility genes for type 2 diabetes (previously identified in genome-wide association studies) are correlated with phosphorylation of signaling factors. Red line denotes positive correlation and green line denotes negative correlation.

SNPs in *STAT3* and *p70S6K* show allelic association with insulin-induced expression changes of *ARL4A* and *NCBP1*, respectively.

### Insulin-responsive kinase–gene interactions

Upon activation, kinases such as AKT and ERK regulate cellular functions through their target genes. Some of the kinase–gene relationships are known, while others are yet to be determined. Since we followed the response of the cells from insulin receptor binding through kinase activations to gene expression changes and these responses showed extensive individual variability, we were poised to infer connections between kinases and the target genes. We determined the correlations between insulin-induced changes in phosphorylation of signaling proteins and the expression of responsive genes. The activation of the kinases is correlated with the expression levels of many genes, including susceptibility genes of type 2 diabetes, such as *THADA*, *CENTD2* and *VEGFA* (Zeggini *et al*, 2008; Voight *et al*, 2010; Fig6B). These correlated kinase–gene pairs (*r* > 0.4) are included in Dataset EV2.

If kinase activity affects gene expression, then the genetic determinants that regulate kinase activities should also influence the expression levels of the target genes. We had found DNA variants that act in *cis* to influence kinase phosphorylation; next, we asked whether these variants have *trans*-effects on gene expression. We analyzed associations between DNA variants within *STAT3*, *p70S6K* and *ERK1/2* and gene expression responses that were correlated with activation of the corresponding kinases. As expected, there were significant allelic associations with changes in expression levels of the corresponding target genes (Table1; nominal *P* < 10^−4^). As shown in Fig6C, T-allele at rs6503697 (chr17:42349561), a SNP in *STAT3* that is associated with higher insulin-induced phosphorylation of STAT3, is associated with higher induction of ADP-ribosylation factor-like 4A (*ARL4A*). Similarly, we found allelic association at rs1292034 (chr17:59912499) in p70S6K with its phosphorylation level in *cis*, and the expression level of *NCBP1* in *trans*. NCBP1 (also known as CBP80) is a subunit of the cap-binding complex that binds to the 5′ cap of pre-mRNAs and is involved in RNA processing and translation initiation. Activated p70S6K following insulin stimulation has been shown to interact with NCBP1 complex and enhance translation efficiency of transcripts (Ma *et al*, 2008). Another example is the sequence variants in ERK that are associated with its insulin-induced phosphorylation in *cis*, and in *trans* the expression levels of several genes such as *DEDD* and *BFAR* that are involved in cell death.

**Table 1:** *Cis*-SNPs that affect kinase activity are associated with gene expression levels in *trans*

SNP	Chr	Position (GRCh38)	Nearby gene	Reference allele	Associated genes	*P*-value
rs11865086	16	30119172	*ERK*	C	*ANGEL1*	7.6 × 10^−4^
rs2005219	16	30129937	*ERK*	A	*BFAR*	9.1 × 10^−5^
rs1292034	17	59912499	*p70S6K*	G	*NCBP1*	2.2 × 10^−5^
rs1292034	17	59912499	*p70S6K*	G	*INF2*	1.2 × 10^−4^
rs1292034	17	59912499	*p70S6K*	G	*FADS1*	3.3 × 10^−4^
rs1292034	17	59912499	*p70S6K*	G	*FADS1*	6.0 × 10^−4^
rs1292034	17	59912499	*p70S6K*	G	*PMAIP1*	8.2 × 10^−4^
rs180515	17	59946914	*p70S6K*	G	*RANBP10*	7.5 × 10^−4^
rs180519	17	59938910	*p70S6K*	G	*NCBP1*	2.2 × 10^−5^
rs180519	17	59938910	*p70S6K*	G	*INF2*	3.1 × 10^−4^
rs180519	17	59938910	*p70S6K*	G	*FADS1*	6.8 × 10^−4^
rs180519	17	59938910	*p70S6K*	G	*KIAA0090*	7.3 × 10^−4^
rs180519	17	59938910	*p70S6K*	G	*FADS1*	8.1 × 10^−4^
rs8071475	17	59896559	*p70S6K*	C	*ACSL3*	8.5 × 10^−4^
rs2293152	17	42329511	*STAT3*	G	*CHST1*	6.1 × 10^−4^
rs6503695	17	42347515	*STAT3*	C	*ARL4A*	2.0 × 10^−5^
rs6503695	17	42347515	*STAT3*	C	*UCHL3*	3.8 × 10^−4^
rs6503695	17	42347515	*STAT3*	C	*JUN*	7.1 × 10^−4^
rs6503695	17	42347515	*STAT3*	C	*KPNA1*	9.0 × 10^−4^
rs6503697	17	42349561	*STAT3*	T	*ARL4A*	2.0 × 10^−5^
rs6503697	17	42349561	*STAT3*	T	*KPNA1*	4.6 × 10^−4^
rs6503697	17	42349561	*STAT3*	T	*UCHL3*	6.4 × 10^−4^

### Insulin-induced activation of ERK affects gene expression and cell growth

**Figure 7:**
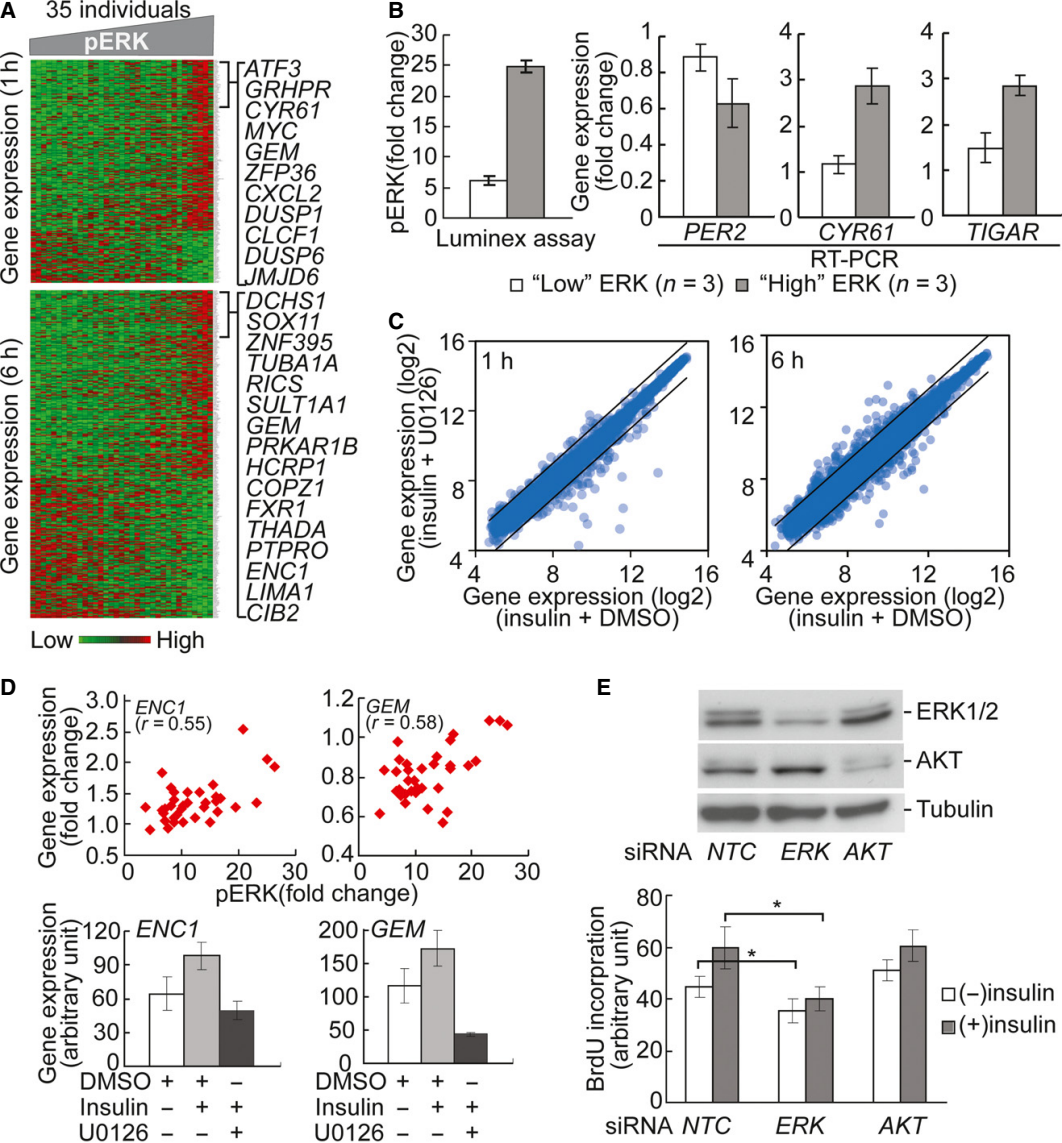
Individual variation in kinase signaling is propagated to downstream processes The heatmap shows the expression levels of genes that are correlated (*r* > 0.4) with phosphorylation of ERK at 1 and 6 h. Columns in the heatmap are sorted according to phosphorylation level of ERK. Similar correlations between genes and other signaling factors were found (data not shown).

To focus on one of the kinase–gene clusters identified from the above analysis, we examined ERK and the genes whose expression levels are correlated with its activation following insulin treatment. The expression levels of 203 genes at one hour and 295 genes at 6 h following insulin treatment are highly correlated (*r*  > 0.4) with ERK phosphorylation. Fig7A shows the expression levels of these genes in our subjects. The genes are significantly enriched for involvement in cell cycle (*CDK10*,*FZR1*,*KIF15*), apoptosis (*BAD*, *FAS*,*PAWR*) and gene expression regulation (*MYC*, *PML*,*SIRT1*). ERK activation explains at least 16% and as much as 44% of individual variability in insulin-induced changes in expression of these genes. To follow up these results, we measured the gene expression levels of *PER2*, *CYR61* and *TIGAR* in individuals with the highest and those with the lowest ERK induction using quantitative PCR. The results validated that expression levels are significantly different between individuals with high and low levels of ERK induction (*P*  < 0.05, *t*-test; Fig7B). *PER2* and other genes in the CLOCK pathways have been shown to play key roles in metabolism (Turek *et al*, 2005; Weber *et al*, 2006; Marcheva *et al*, 2010).

Quantitative PCR validates that insulin-induced gene expression is significantly different (*P *< 0.05, *t*-test) between individuals with high and low ERK phosphorylation. Insulin-induced ERK phosphorylation in 3 individuals with “high” and “low” induction was quantified by Luminex assay. Gene expression levels were measured using quantitative RT–PCR. Error bars represent SEM among 3 individuals of each group.

U0126 treatment led to change in expression level of genes that are correlated with ERK phosphorylation. Lines in scatter plots indicate threshold of 2-fold change.

Two examples of genes regulated by ERK. Changes in expression levels of *ENC1* and *GEM1* are correlated with ERK phosphorylation. Following ERK inhibition by U0126, their expression response diminished. Cells from 4 individuals were treated and average values are shown. Error bars represent SEM among 4 individuals.

Knockdown of *ERK1* inhibits cell cycle progression. Western blot shows that siRNA knockdown of *ERK1* and *AKT* resulted in reduction of protein level. NTC, negative control siRNA. BrdU incorporation assay shows that *ERK1* knockdown reduced insulin-induced DNA synthesis. In contrast, *AKT* knockdown did not affect BrdU incorporation. Average values from 4 individuals are shown. Error bars represent SEM. *P < 0.02; paired *t*-test. Source data are available online for this figure.

While there were significant correlations between activation of signaling factors and gene expression levels, the correlation alone does not imply that ERK phosphorylation regulates expression of these genes. Thus, we treated the cells with an inhibitor of ERK phosphorylation, U0126 (Favata *et al*, 1998), to examine the specific effect of ERK on gene expression. We found that U0126 treatment led to changes in the expression levels of insulin-responsive genes that co-vary with ERK (Fig7C). For instance, U0126 repressed the expression of *ENC1* and *GEM* whose expression levels were positively correlated with ERK phosphorylation (*r* = 0.55 and 0.58, respectively; Fig7D). *ENC1* encodes an actin-binding protein which is involved in adipocyte differentiation (Zhao *et al*, 2000). Its overexpression is also associated with various cancers (Fujita *et al*, 2001; Hammarsund *et al*, 2004; Durand *et al*, 2011). The positive correlation between *ENC1* expression and ERK phosphorylation in response to insulin suggests a possible mechanism for higher cancer risk in patients with insulin resistance (Arcidiacono *et al*, 2012). *GEM* encodes an RGK family of GTPase, which regulates voltage-dependent Ca^2+^ channels. It has been shown that Gem-null mice were glucose intolerant and have impaired insulin secretion (Gunton *et al*, 2012). Here we showed that gene expression of *GEM* is regulated by insulin-stimulated ERK phosphorylation, suggesting a feedback pathway between insulin secretion and insulin signaling.

Among the genes that correlate with ERK phosphorylation are ones that regulate the cell cycle. This is consistent with previous reports that ERK signaling affects insulin-induced cell proliferation (Samii *et al*, 1998; Conejo & Lorenzo, 2001). We used short interfering RNA (siRNA) to knockdown *ERK* expression in fibroblasts, then treated the cells with insulin and measured cell cycle progression by BrdU incorporation (Fig.7E). The reduction of ERK protein levels resulted in a significant decrease (*P* < 0.02, *t*-test) of BrdU incorporation. In contrast, siRNA knockdown of *AKT* did not affect BrdU incorporation, suggesting that ERK pathway plays more prominent roles in insulin-induced cell proliferation. This further supports the biological significance of individual differences in ERK activation and its contribution to variation in insulin sensitivity among individuals.

## Discussion

In summary, our results reveal extensive individual variation in insulin-induced signaling. To maintain proper functions, cells must respond to cellular and environmental inputs. Signal transduction is the key step in transmitting environmental cues to cellular processes. We found that insulin-induced signal transduction through proteins such as ERK, AKT and p70S6K varies greatly among individuals. We showed that like gene expression (Cheung & Spielman, 2009; Smirnov *et al*, 2009), there is a genetic component to the individual variation in signal transduction. Induction of the signaling proteins differs in individuals with different polymorphic forms of the proteins. The variability in signaling response to insulin leads to differences in expression of genes which direct downstream cellular processes. Specifically, we found that individual differences in insulin-induced ERK phosphorylation resulted in variation in expression levels of genes that regulate metabolic and mitogenic processes, and downstream cell cycle progression. Our study was carried out using samples from newborns. It would be interesting to carry out similar analyses in adults which would provide additional information on how environmental factors, such as adult diets, influence the genetic effects.

By identifying signal transduction as a contributing factor to individual differences in insulin response, our findings offer an opportunity to tailor pharmacologic correction of insulin resistance. Identifications of the roles of abnormal kinase activities in cancer have promoted the development of protein kinase inhibitors such as ST1571 (Gleevec; Druker *et al*, 1996) and ZD-1839 (Iressa; Ciardiello *et al*, 2000) as therapeutics. Here, we show that kinase modulators are potential therapeutics for insulin resistance. Current pharmacologic treatment of diabetes relies on two main classes of drugs, insulin mimetics and insulin sensitizers. In many patients, these drugs do not provide optimal hyperglycemic control. Our results suggest that some of these patients may carry genetic variants that lower their signaling response to insulin and its mimetics. Knowledge of the specific kinases that are more or less active in patients will guide more tailored treatments; for instance, patients can be given the modulators that stimulate their less efficient kinases.

Genome sequencing is already used increasingly for diagnostic purposes. The extension of genetics in medicine from diagnosis to treatment relies on knowledge of the molecular effects of sequence variants. As our ability to carry out functional analyses advances, it is now becoming feasible to assess the influence of DNA variants on cellular phenotypes. As we demonstrate here, it is possible to identify sequence variants that affect insulin response and the pathways that these variants act on. A combination of genetics, functional genomics and cell biology will improve clinical medicine by enabling not only more precise diagnosis but also targeted therapy.

## Materials and Methods

### Cell culture

Foreskin tissues were collected from circumcisions of 35 healthy and unrelated 3-day-old newborns (anonymous donors at the Hospital of the University of Pennsylvania). Our Institutional Review Board approved our samples as exemption from human subject research, and no informed consent was required. The samples are discarded foreskin samples from anonymous donors that cannot be traced. Primary fibroblasts were isolated from these foreskin tissues. Briefly, the tissues were sectioned and epidermis removed. The remaining dermis was incubated in 1 ml of 3 mg/ml collagenase (Roche) in HBSS buffer with calcium and magnesium (Mediatech) at 37°C for 30 min. One milliliter of 0.5% trypsin/EDTA (Invitrogen) was then added, and the tissue was incubated at 37°C for 10 min. Trypsin was inactivated by adding 1 ml MEM medium (Invitrogen) with 10% fetal bovine serum (Hyclone); then, the undigested tissue pieces were removed, and fibroblasts were collected from suspension by centrifugation. Passage-1 fibroblasts were subsequently cultured at 37°C in 5% CO_2_ in MEM medium supplemented with 10% fetal bovine serum, 2 mM L-glutamine, 100 U/ml penicillin and 100 μg/ml streptomycin and were passaged every 3 days.

To minimize technical variability, fibroblasts of the same passage (passage 4) from the 35 individuals were treated and harvested in one batch for signaling assays and microarray experiments. Fibroblasts were seeded at a density of 3 × 10^4^/cm^2^ and incubated for 24 h at 37°C to ∼70–80% confluency. Cells were then washed once with phosphate-buffered saline and incubated for another 18 h in serum-free medium.

### Luminex signaling assay

Fibroblasts of passage 4 were serum-starved for 18 h and treated with mock or 100 nM insulin for 10 min. Cells were then lysed in 1× MILLIPLEX lysis buffer (Millipore). Phosphorylation of signaling factors was measured using the Luminex Multi-pathway Signaling Kits (Millipore; Bio-Rad): INSRβ (Tyr1146), IRS1 (Ser636/Ser639), MEK1 (Ser217/Ser212), AKT (Ser473), ERK1/2 (Thr185/Tyr187), STAT3 (Ser727), JNK (Thr183/Tyr185), p70 S6 kinase (Thr412), IkBalpha (Ser32), STAT5A/B (Tyr694/699), CREB (Ser133), p38 (Thr180/Tyr182), GSK-3α/β (Ser21/Ser9), c-Jun (Ser63) and HSP27 (Ser78). Total protein levels of p38, ERK1/2, AKT, STAT3, JNK, p70 S6 kinase and CREB were measured using the Luminex assay (Millipore). GAPDH protein level was measured as a loading control for total protein input. Cells from each individual were treated and assayed twice independently. Fold change was calculated by taking the ratio between phosphorylation levels from insulin-treated cells and those from mock-treated cells. Fold difference in total protein levels was calculated by taking the ratio between a given individual and the individual with the lowest protein level. We report the average fold changes from the biological replicates.

As a measurement of individual variation relative to technical variability, for each signaling protein, we calculated variance ratio, the ratio between variance of insulin-induced phosphorylation among individuals and the variance within individuals (replicates). For all signaling proteins, the variance among individuals was significantly higher than that within replicates (*P* < 0.05).

### Gene expression measurement by microarray

Fibroblasts of passage 4 from 35 individuals were serum-starved for 18 h and treated with 100 nM insulin for 0, 1 and 6 h. In the ERK inhibition studies, cells from 4 individuals were treated with 10 μM U0126 (Cell Signaling) or DMSO (Sigma-Aldrich) for 1 h before 100 nM insulin or ddH2O (mock treatment) was added. Total RNA was extracted using RNeasy Micro Kit (QIAGEN). cDNA was synthesized using oligo (dT) primer following the manufacturer’s protocol (TaqMan Reverse Transcription Reagents; Applied Biosystems). Real-time PCR was performed on 7900HT Real-time PCR System using Power SYBR Green Master Mix (Applied Biosystems). PCRs were performed in duplicates and average fold changes of 4 individuals are shown, along with standard error of the mean (SEM). For microarrays, cRNA was amplified, labeled and hybridized to Affymetrix Human U133A 2.0 arrays. The array data were analyzed using Expression Console software (Affymetrix). Expression values were scaled using MAS5 algorithm and log2-transformed. A total of 11,845 probesets were determined as “expressed” in fibroblasts (80% of the samples based on presence call values). To identify probesets whose expression levels changed among 3 time points, we carried out analysis of variance with correction for multiple testing (*P* < 10^−6^; ANOVA).

### Stable isotope labeling by amino acids in cell culture (SILAC) and mass spectrometry

Primary fibroblasts from one individual was cultured in D-MEM supplemented with 10% dialyzed fetal bovine serum (Invitrogen), 1× L-glutamine, 100 U/ml penicillin and 100 μg/ml streptomycin, (MS10030, Invitrogen). For “light” medium, L-arginine and L-lysine were added; for “heavy” medium, [U-^13^C6]-L-lysine HCl and [U-^13^C6, ^15^N4]-L-arginine were added. Cells were passaged six times in each medium, and whole cell extracts collected from equal number of cells were mixed 1:1 and fractioned using GelC method. One fraction was analyzed by mass spectrometry to confirm “heavy” isotope labeling efficiency is > 96%. Fibroblasts were then serum-starved for 18 h, and cells in heavy medium were treated with 100 nM insulin for 6 h before harvesting. Whole cell lysates from equal number of cells in light and heavy medium were mixed 1:1, separated into 23 fractions by GelC, trypsin-digested, and extracted tryptic peptides from each fraction were injected onto a nanocapillary reverse-phase column (75-μm column terminating in a nanospray 15-μm tip) directly coupled to a LTQ-Orbitrap mass spectrometer (Thermo Scientific). The MS/MS data were acquired using a top six method. Each fraction was injected and analyzed in duplicates.

Raw files acquired from LC/MS-MS were analyzed by MaxQuant (version 1.1.1.25; Cox & Mann, 2008) using the following parameters: peptide FDR 0.01; protein FDR 0.01; minimal peptide length = 6; variable modifications: oxidation (M), acetyl (protein N-term); fixed modifications: carbamidomethyl (C); special AAs: KR; MS/MS tol. (HCD), 20 ppm; decoy search enabled; and database: ipi.HUMAN.v3.68.fasta. Only protein groups with at least two unique peptides identified were retained for further analysis. Significance of normalized H/L protein ratio was calculated taking into account peptide intensity (Significance B, MaxQuant; Cox *et al*, 2009).

### Immunoprecipitation and Western blot

Primary fibroblasts were serum-starved for 18 h before treatment with 100 nM insulin for 5 min. Cells were lysed in 1× lysis buffer (20 mM Tris–HCl (pH 7.5), 150 mM NaCl, 1 mM Na_2_EDTA, 1 mM EGTA, 1% Triton; Cell Signaling) supplemented with 1× COMPLETE protease inhibitors (Roche) and 1× phosphatase inhibitors I and II (Sigma-Aldrich). Cell lysates containing 150 μg of total protein were incubated with 5 μg α-INSR antibody (Cell Signaling, 3025), or 5 μg α-IGF1R antibody (Cell Signaling, 3018) at 4°C overnight. Immunocomplex was pulled down using Protein A-Sepharose (GE Healthcare). Input and immunoprecipitation samples were analyzed by Western blot using α-phosphotyrosine antibody (1:1,000; Millipore, 4G10 Platinum), or the above α-INSR (1:1,000) and α-IGF1R (1:1,000) antibodies. Twenty percent of input lysates were loaded as a control.

### siRNA knockdown and BrdU assay

Primary fibroblasts from 4 individuals were transfected with 100 nM pooled siRNA specific for ERK1/2 or AKT (Cell Signaling, #6560; #6211) or negative control siRNA, respectively, using lipofectamine RNAiMAX (Invitrogen). Western blot analyses with antibodies specific for ERK1/2 and AKT (Cell Signaling) were used to confirm the reduction of protein levels following gene knockdown. Twenty-four hours post-transfection, cells were serum-starved for 18 h and treated with 100 nM insulin for 22 h. BrdU was then added to medium, and cells were incubated for another 2 h before cells were fixed and assayed using the Cell Proliferation ELISA kit (Roche) following manufacture’s protocol. The average values from 4 individuals were shown.

### Association analysis

SNPs within the genes coding signaling proteins (10 kb upstream of 5′ UTR to 10 kb downstream of 3′ UTR; minor allele frequency > 0.05) were identified based on dbSNP. Genome location of each SNP is based on the latest build in dbSNP (GRCh38/hg38). DNA samples from the 35 individuals were genotyped using ABI TaqMan SNP genotyping assays on the 7900HT real-time PCR system (Applied Biosystems). Association analysis was carried out using PLINK (version 1.07; Purcell *et al*, 2007). Phosphorylation of each protein, as dependent variable, was regressed on SNP genotypes (coded 0, 1 and 2). One outlier in signaling assay was removed; thus, only 34 individuals were included in the following analysis. We also compared the results with those from non-parametric analysis of Kruskal–Wallis test, and similar results were obtained. SNPs within genes are correlated; to adjust for this correlation in correction for multiple testing, we used the method developed by Nyholt (2004).

### Data availability

Data were deposited in Gene Expression Omnibus (GSE21891) and PeptideAtlas (Dataset Identifier: PASS00688, Dataset Password: YP6525d).

## Supporting Information

**Expanded View Figures PDF**

**Dataset EV1**

**Dataset EV2**

**Review Process File**

**Source Data for Figure 4**

**Source Data for Figure 7E**

**Source Data for Expanded View**

## References

[b1] Aalto-Setälä K, Fisher EA, Chen X, Chajek-Shaul T, Hayek T, Zechner R, Walsh A, Ramakrishnan R, Ginsberg HN, Breslow JL (1992). Mechanism of hypertriglyceridemia in human apolipoprotein (apo) CIII transgenic mice. Diminished very low density lipoprotein fractional catabolic rate associated with increased apo CIII and reduced apo E on the particles. J Clin Invest.

[b2] Arcidiacono B, Iiritano S, Nocera A, Possidente K, Nevolo MT, Ventura V, Foti D, Chiefari E, Brunetti A (2012). Insulin resistance and cancer risk: an overview of the pathogenetic mechanisms. J Diabetes Res.

[b3] Ashburner M, Ball CA, Blake JA, Botstein D, Butler H, Cherry JM, Davis AP, Dolinski K, Dwight SS, Eppig JT, Harris MA, Hill DP, Issel-Tarver L, Kasarskis A, Lewis S, Matese JC, Richardson JE, Ringwald M, Rubin GM, Sherlock G (2000). Gene ontology: tool for the unification of biology. The Gene Ontology Consortium. Nat Genet.

[b4] Borisov N, Aksamitiene E, Kiyatkin A, Legewie S, Berkhout J, Maiwald T, Kaimachnikov NP, Timmer J, Hoek JB, Kholodenko BN (2009). Systems-level interactions between insulin–EGF networks amplify mitogenic signaling. Mol Syst Biol.

[b5] Bost F, Aouadi M, Caron L, Binétruy B (2005). The role of MAPKs in adipocyte differentiation and obesity. Biochimie.

[b6] Bouatia-Naji N, Rocheleau G, Van Lommel L, Lemaire K, Schuit F, Cavalcanti-Proença C, Marchand M, Hartikainen A-L, Sovio U, De Graeve F, Rung J, Vaxillaire M, Tichet J, Marre M, Balkau B, Weill J, Elliott P, Jarvelin M-R, Meyre D, Polychronakos C (2008). A polymorphism within the G6PC2 gene is associated with fasting plasma glucose levels. Science.

[b7] Boulton TG, Nye SH, Robbins DJ, Ip NY, Radziejewska E, Morgenbesser SD, DePinho RA, Panayotatos N, Cobb MH, Yancopoulos GD (1991). ERKs: a family of protein-serine/threonine kinases that are activated and tyrosine phosphorylated in response to insulin and NGF. Cell.

[b8] Burgering BM, Coffer PJ (1995). Protein kinase B (c-Akt) in phosphatidylinositol-3-OH kinase signal transduction. Nature.

[b9] Carlson LA, Ballantyne D (1976). Changing relative proportions of apolipoproteins CII and CIII of very low density lipoproteins in hypertriglyceridaemia. Atherosclerosis.

[b10] Chen M, Breslow JL, Li W, Leff T (1994). Transcriptional regulation of the apoC-III gene by insulin in diabetic mice: correlation with changes in plasma triglyceride levels. J Lipid Res.

[b11] Cheung VG, Spielman RS (2009). Genetics of human gene expression: mapping DNA variants that influence gene expression. Nat Rev Genet.

[b12] Ciardiello F, Caputo R, Bianco R, Damiano V, Pomatico G, De Placido S, Bianco AR, Tortora G (2000). Antitumor effect and potentiation of cytotoxic drugs activity in human cancer cells by ZD-1839 (Iressa), an epidermal growth factor receptor-selective tyrosine kinase inhibitor. Clin Cancer Res.

[b13] Clausen JO, Borch-Johnsen K, Ibsen H, Bergman RN, Hougaard P, Winther K, Pedersen O (1996). Insulin sensitivity index, acute insulin response, and glucose effectiveness in a population-based sample of 380 young healthy Caucasians. Analysis of the impact of gender, body fat, physical fitness, and life-style factors. J Clin Invest.

[b14] Conejo R, Lorenzo M (2001). Insulin signaling leading to proliferation, survival, and membrane ruffling in C2C12 myoblasts. J Cell Physiol.

[b15] Cox J, Mann M (2008). MaxQuant enables high peptide identification rates, individualized p.p.b.-range mass accuracies and proteome-wide protein quantification. Nat Biotechnol.

[b16] Cox J, Matic I, Hilger M, Nagaraj N, Selbach M, Olsen JV, Mann M (2009). A practical guide to the MaxQuant computational platform for SILAC-based quantitative proteomics. Nat Protoc.

[b17] De Godoy LMF, Olsen JV, Cox J, Nielsen ML, Hubner NC, Fröhlich F, Walther TC, Mann M (2008). Comprehensive mass-spectrometry-based proteome quantification of haploid versus diploid yeast. Nature.

[b18] Mahajan A, Go MJ, Zhang W, Below JE, Gaulton KJ, Ferreira T, Horikoshi M, Johnson AD, Ng MCY, Prokopenko I, Saleheen D, Wang X, Zeggini E, Abecasis GR, Adair LS, DIAbetes Genetics Replication And Meta-analysis (DIAGRAM) Consortium, Asian Genetic Epidemiology Network Type 2 Diabetes (AGEN-T2D) Consortium, South Asian Type 2 Diabetes (SAT2D) Consortium, Mexican American Type 2 Diabetes (MAT2D) Consortium, Type 2 Diabetes Genetic Exploration by Next-generation sequencing in multi-Ethnic Samples (T2D-GENES) Consortium (2014). Genome-wide trans-ancestry meta-analysis provides insight into the genetic architecture of type 2 diabetes susceptibility. Nat Genet.

[b19] Drong AW, Lindgren CM, McCarthy MI (2012). The genetic and epigenetic basis of type 2 diabetes and obesity. Clin Pharmacol Ther.

[b20] Druker BJ, Tamura S, Buchdunger E, Ohno S, Segal GM, Fanning S, Zimmermann J, Lydon NB (1996). Effects of a selective inhibitor of the Abl tyrosine kinase on the growth of Bcr-Abl positive cells. Nat Med.

[b21] Dunaif A, Xia J, Book CB, Schenker E, Tang Z (1995). Excessive insulin receptor serine phosphorylation in cultured fibroblasts and in skeletal muscle. A potential mechanism for insulin resistance in the polycystic ovary syndrome. J Clin Invest.

[b22] Durand J, Lampron A, Mazzuco TL, Chapman A, Bourdeau I (2011). Characterization of differential gene expression in adrenocortical tumors harboring β-catenin (CTNNB1) mutations. J Clin Endocrinol Metab.

[b23] Eckardt K, May C, Koenen M, Eckel J (2007). IGF-1 receptor signalling determines the mitogenic potency of insulin analogues in human smooth muscle cells and fibroblasts. Diabetologia.

[b24] Engelman JA, Luo J, Cantley LC (2006). The evolution of phosphatidylinositol 3-kinases as regulators of growth and metabolism. Nat Rev Genet.

[b25] Favata MF, Horiuchi KY, Manos EJ, Daulerio AJ, Stradley DA, Feeser WS, Van Dyk DE, Pitts WJ, Earl RA, Hobbs F, Copeland RA, Magolda RL, Scherle PA, Trzaskos JM (1998). Identification of a novel inhibitor of mitogen-activated protein kinase kinase. J Biol Chem.

[b26] Frittitta L, Spampinato D, Solini A, Nosadini R, Goldfine ID, Vigneri R, Trischitta V (1998). Elevated PC-1 content in cultured skin fibroblasts correlates with decreased *in vivo* and *in vitro* insulin action in nondiabetic subjects: evidence that PC-1 may be an intrinsic factor in impaired insulin receptor signaling. Diabetes.

[b27] Fujita M, Furukawa Y, Tsunoda T, Tanaka T, Ogawa M, Nakamura Y (2001). Up-regulation of the ectodermal-neural cortex 1 (ENC1) gene, a downstream target of the beta-catenin/T-cell factor complex, in colorectal carcinomas. Cancer Res.

[b28] Ginsberg HN, Le NA, Goldberg IJ, Gibson JC, Rubinstein A, Wang-Iverson P, Norum R, Brown WV (1986). Apolipoprotein B metabolism in subjects with deficiency of apolipoproteins CIII and AI. Evidence that apolipoprotein CIII inhibits catabolism of triglyceride-rich lipoproteins by lipoprotein lipase *in vivo*. J Clin Invest.

[b29] Gunton JE, Sisavanh M, Stokes RA, Satin J, Satin LS, Zhang M, Liu SM, Cai W, Cheng K, Cooney GJ, Laybutt DR, So T, Molero J-C, Grey ST, Andres DA, Rolph MS, Mackay CR (2012). Mice deficient in GEM GTPase show abnormal glucose homeostasis due to defects in beta-cell calcium handling. PLoS ONE.

[b30] Gygi SP, Rochon Y, Franza BR, Aebersold R (1999). Correlation between protein and mRNA abundance in yeast. Mol Cell Biol.

[b31] Hammarsund M, Lerner M, Zhu C, Merup M, Jansson M, Gahrton G, Kluin-Nelemans H, Einhorn S, Grandér D, Sangfelt O, Corcoran M (2004). Disruption of a novel ectodermal neural cortex 1 antisense gene, ENC-1AS and identification of ENC-1 overexpression in hairy cell leukemia. Hum Mol Genet.

[b32] Hansen T, Andersen CB, Echwald SM, Urhammer SA, Clausen JO, Vestergaard H, Owens D, Hansen L, Pedersen O (1997). Identification of a common amino acid polymorphism in the p85alpha regulatory subunit of phosphatidylinositol 3-kinase: effects on glucose disappearance constant, glucose effectiveness, and the insulin sensitivity index. Diabetes.

[b33] International Diabetes Federation (2013). IDF Diabetes Atlas.

[b34] Ito Y, Azrolan N, O’Connell A, Walsh A, Breslow JL (1990). Hypertriglyceridemia as a result of human apo CIII gene expression in transgenic mice. Science.

[b35] Jiao P, Feng B, Li Y, He Q, Xu H (2013). Hepatic ERK activity plays a role in energy metabolism. Mol Cell Endocrinol.

[b36] Kim Y-B, Ciaraldi TP, Kong A, Kim D, Chu N, Mohideen P, Mudaliar S, Henry RR, Kahn BB (2002). Troglitazone but not metformin restores insulin-stimulated phosphoinositide 3-kinase activity and increases p110beta protein levels in skeletal muscle of type 2 diabetic subjects. Diabetes.

[b37] Kislinger T, Cox B, Kannan A, Chung C, Hu P, Ignatchenko A, Scott MS, Gramolini AO, Morris Q, Hallett MT, Rossant J, Hughes TR, Frey B, Emili A (2006). Global survey of organ and organelle protein expression in mouse: combined proteomic and transcriptomic profiling. Cell.

[b38] Li WW, Dammerman MM, Smith JD, Metzger S, Breslow JL, Leff T (1995). Common genetic variation in the promoter of the human apo CIII gene abolishes regulation by insulin and may contribute to hypertriglyceridemia. J Clin Invest.

[b39] Ma XM, Yoon S-O, Richardson CJ, Jülich K, Blenis J (2008). SKAR links Pre-mRNA splicing to mTOR/S6K1-mediated enhanced translation efficiency of spliced mRNAs. Cell.

[b40] Marcheva B, Ramsey KM, Buhr ED, Kobayashi Y, Su H, Ko CH, Ivanova G, Omura C, Mo S, Vitaterna MH, Lopez JP, Philipson LH, Bradfield CA, Crosby SD, JeBailey L, Wang X, Takahashi JS, Bass J (2010). Disruption of the clock components CLOCK and BMAL1 leads to hypoinsulinaemia and diabetes. Nature.

[b41] Melmed S, Polonsky KS, Larsen PR, Kronenberg HM (2011). Williams Textbook of Endocrinology.

[b42] Nyholt DR (2004). A simple correction for multiple testing for single-nucleotide polymorphisms in linkage disequilibrium with each other. Am J Hum Genet.

[b43] Ong S-E, Blagoev B, Kratchmarova I, Kristensen DB, Steen H, Pandey A, Mann M (2002). Stable isotope labeling by amino acids in cell culture, SILAC, as a simple and accurate approach to expression proteomics. Mol Cell Proteomics.

[b44] Pollin TI, Damcott CM, Shen H, Ott SH, Shelton J, Horenstein RB, Post W, McLenithan JC, Bielak LF, Peyser PA, Mitchell BD, Miller M, O’Connell JR, Shuldiner AR (2008). A null mutation in human APOC3 confers a favorable plasma lipid profile and apparent cardioprotection. Science.

[b45] Porter KE, Turner NA (2009). Cardiac fibroblasts: at the heart of myocardial remodeling. Pharmacol Ther.

[b46] Prudente S, Morini E, Trischitta V (2009). Insulin signaling regulating genes:effect on T2DM and cardiovascular risk. Nat Rev Endocrinol.

[b47] Purcell S, Neale B, Todd-Brown K, Thomas L, Ferreira MAR, Bender D, Maller J, Sklar P, de Bakker PIW, Daly MJ, Sham PC (2007). PLINK: a tool set for whole-genome association and population-based linkage analyses. Am J Hum Genet.

[b48] Samii A, Lopez-Devine J, Wasserman EM, Dalakas MC, Clark K, Grafman J, Hallett M (1998). Normal postexercise facilitation and depression of motor evoked potentials in postpolio patients. Muscle Nerve.

[b49] Schilling EE, Rechler MM, Grunfeld C, Rosenberg AM (1979). Primary defect of insulin receptors in skin fibroblasts cultured from an infant with leprechaunism and insulin resistance. Proc Natl Acad Sci USA.

[b50] Smigielski EM, Sirotkin K, Ward M, Sherry ST (2000). dbSNP: a database of single nucleotide polymorphisms. Nucleic Acids Res.

[b51] Smirnov DA, Morley M, Shin E, Spielman RS, Cheung VG (2009). Genetic analysis of radiation-induced changes in human gene expression. Nature.

[b52] Taylor SI, Roth J, Blizzard RM, Elders MJ (1981). Qualitative abnormalities in insulin binding in a patient with extreme insulin resistance: decreased sensitivity to alterations in temperature and pH. Proc Natl Acad Sci USA.

[b53] Turek FW, Joshu C, Kohsaka A, Lin E, Ivanova G, McDearmon E, Laposky A, Losee-Olson S, Easton A, Jensen DR, Eckel RH, Takahashi JS, Bass J (2005). Obesity and metabolic syndrome in circadian Clock mutant mice. Science.

[b54] Voight BF, Scott LJ, Steinthorsdottir V, Morris AP, Dina C, Welch RP, Zeggini E, Huth C, Aulchenko YS, Thorleifsson G, McCulloch LJ, Ferreira T, Grallert H, Amin N, Wu G, Willer CJ, Raychaudhuri S, McCarroll SA, Langenberg C, Hofmann OM (2010). Twelve type 2 diabetes susceptibility loci identified through large-scale association analysis. Nat Genet.

[b55] Weber F, Hung H-C, Maurer C, Kay SA (2006). Second messenger and Ras/MAPK signalling pathways regulate CLOCK/CYCLE-dependent transcription. J Neurochem.

[b56] Wu JJ, Roth RJ, Anderson EJ, Hong E-G, Lee M-K, Choi CS, Neufer PD, Shulman GI, Kim JK, Bennett AM (2006). Mice lacking MAP kinase phosphatase-1 have enhanced MAP kinase activity and resistance to diet-induced obesity. Cell Metab.

[b57] Zeggini E, Scott LJ, Saxena R, Voight BF, Marchini JL, Hu T, de Bakker PIW, Abecasis GR, Almgren P, Andersen G, Ardlie K, Boström KB, Bergman RN, Bonnycastle LL, Borch-Johnsen K, Burtt NP, Chen H, Chines PS, Daly MJ, Deodhar P (2008). Meta-analysis of genome-wide association data and large-scale replication identifies additional susceptibility loci for type 2 diabetes. Nat Genet.

[b58] Zhao L, Gregoire F, Sul HS (2000). Transient induction of ENC-1, a Kelch-related actin-binding protein, is required for adipocyte differentiation. J Biol Chem.

[b59] Zhou G, Myers R, Li Y, Chen Y, Shen X, Fenyk-Melody J, Wu M, Ventre J, Doebber T, Fujii N, Musi N, Hirshman MF, Goodyear LJ, Moller DE (2001). Role of AMP-activated protein kinase in mechanism of metformin action. J Clin Invest.

