# Supplementary material for: Genetic variation in insulin-induced kinase signaling
Source: Mol Syst Biol. 2015 Jul 22;11(7):820. doi: 10.15252/msb.20156250 (PMC4547848; doi:10.15252/msb.20156250)
Supplement: Supplementary file 1 [file msb0011-0820-sd1.pdf]

Expanded View Figures

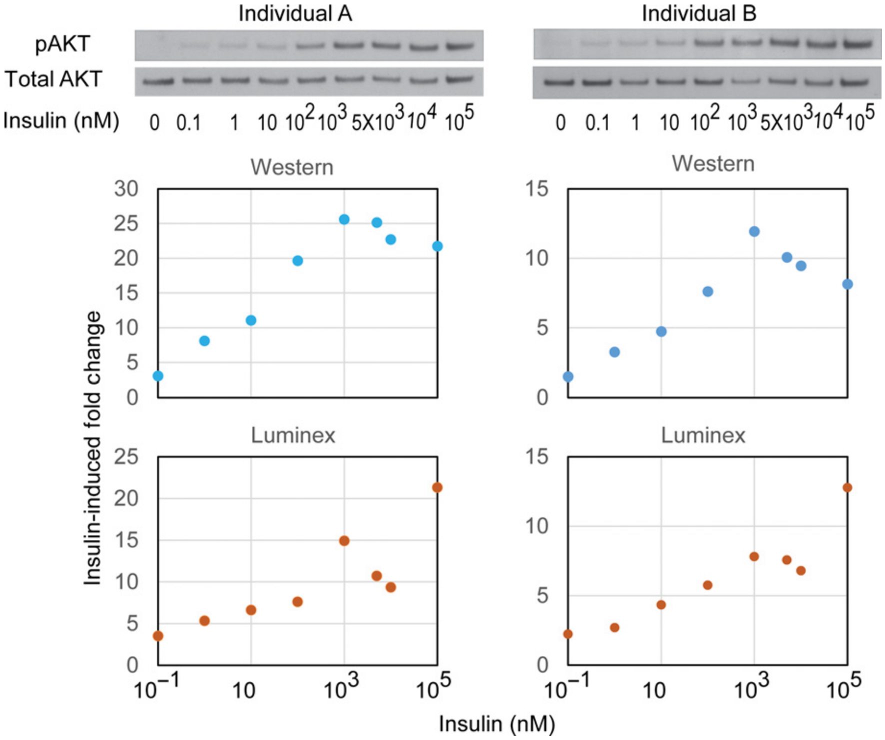

Figure EV1. Dose-dependent insulin response in skin fibroblasts.
Skin fibroblasts from two individuals were treated with a serial dilution of insulin (0.1–10^5 nM) for 10 min. Whole cell lysates were harvested and analyzed by Western blot and Luminex phosphorylation assay in parallel using antibodies against phospho-Ser473 of AKT or total AKT. Western blot signal was quantified using ImageJ. Fold change of AKT phosphorylation following insulin treatment is shown.

Source data are available online for this figure.

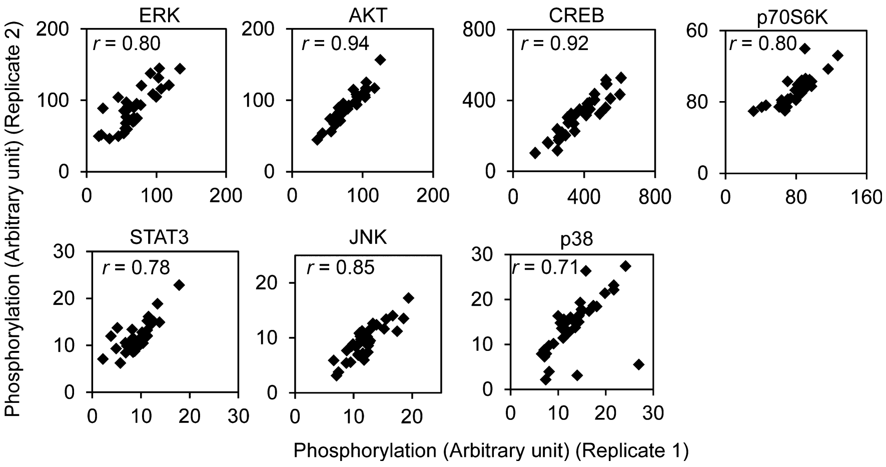

Figure EV2. Luminex assay results from biological duplicates are highly similar.
Fibroblasts from 35 individuals were seeded in duplicate wells, serum-starved and treated with 100 nM insulin for 10 min. Cells were lysed and phosphorylation of each target was measured by Luminex assay.

© 2015 The Authors

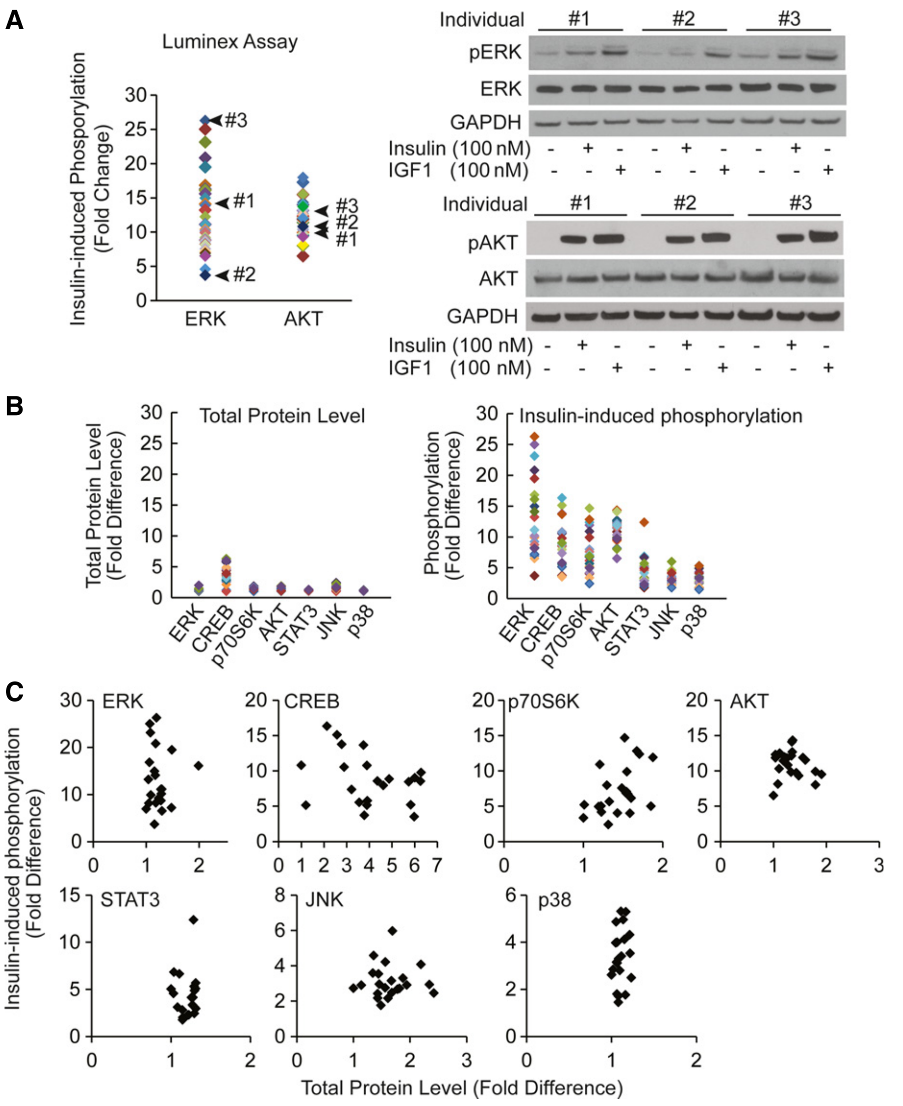

Figure EV3. Luminex phosphorylation measurements are reproducible and have broad dynamic range.

A Individual difference in insulin-induced phosphorylation of ERK measured by Luminex assay is validated by Western blot. Skin fibroblasts from the indicated 3 individuals were treated with 100 nM insulin in duplicates. Cells were harvested and assayed using Luminex phosphorylation assay or Western blot using the same antibodies. Cells were treated with 100 nM IGF1 as a positive control. Total protein levels of AKT or ERK did not change before and after insulin treatment.

B Compared to differences in phosphorylation levels, total protein levels are not as variable across individuals. Total protein levels or insulin-induced phosphorylation levels of 7 signaling factors shown in Fig 5A were measured in 20 individuals by Luminex assay. Average value of biological duplicates is shown.

C Fluctuation in total protein levels does not contribute to variation in phosphorylation levels induced by insulin. The same data as in (B) were re-plotted to show there is no correlation between phosphorylation levels and total protein levels.

Source data are available online for this figure.

 © 2015 The Authors

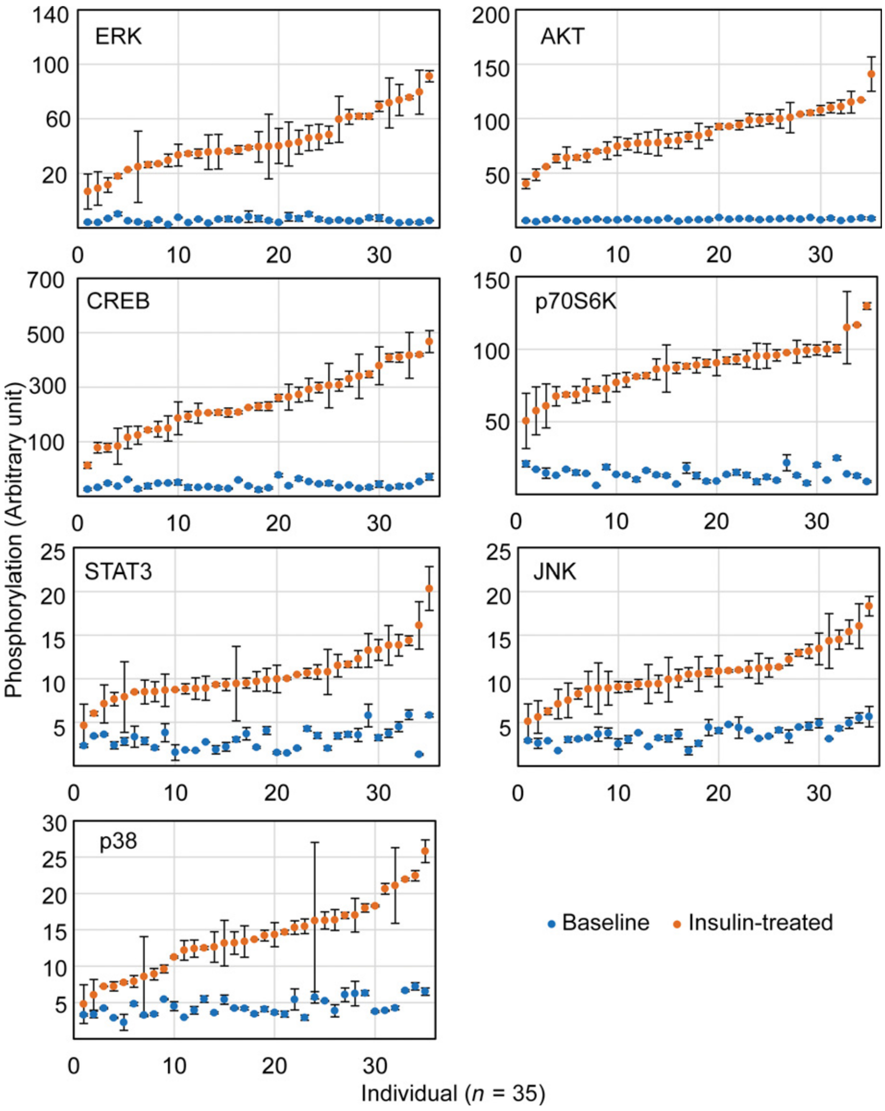

Figure EV4. Phosphorylation levels of signaling factors after insulin treatment do not show significant correlations with those at baseline.
Phosphorylation of each signaling factor at baseline or after insulin treatment from the same individual is shown in the same column. Variation among individuals in insulin-induced phosphorylation is significantly greater than that within replicated assays ($P < 0.05$; ANOVA) for all seven signaling factors. The average phosphorylation level of replicates is shown. Error bars represent SEM of biological replicates.

© 2015 The Authors
