# Supplementary material for: Genetic variation in insulin-induced kinase signaling
Source: Mol Syst Biol. 2015 Jul 22;11(7):820. doi: 10.15252/msb.20156250 (PMC4547848; doi:10.15252/msb.20156250)
Supplement: Supplementary file 4 [file msb0011-0820-sd4.pdf]

Genetic Variation in Insulin-Induced Kinase Signaling

Isabel Xiaorong Wang, Girish Ramrattan, Vivian G. Cheung

Corresponding author: Vivian G. Cheung, Howard Hughes Medical Institute; University of Michigan

Review timeline:

Submission date:	28 July 2014
Editorial Decision:	3 September 2014
New Submission date:	23 April 2015
Editorial Decision:	11 May 2015
Revision received:	27 May 2015
Editorial Decision:	16 June 2015
Revision received:	18 June 2015
Accepted:	26 June 2015

Editor: Maria Polychronidou

Transaction Report:

(Note: With the exception of the correction of typographical or spelling errors that could be a source of ambiguity, letters and reports are not edited. The original formatting of letters and referee reports may not be reflected in this compilation.)

1st Editorial Decision 3 September 2014

Thank you again for submitting your work to Molecular Systems Biology. We have now heard back from the three referees who agreed to evaluate your manuscript. As you will see from the reports below, the referees raise substantial concerns on your work, which, I am afraid to say, preclude its publication in Molecular Systems Biology.

While reviewer #1 is overall positive, reviewers #2 and #3 raise significant concerns regarding the analysis and interpretation of the data and they are not convinced about the validity of the main conclusions. Considering the substantial concerns raised and the overall low level of support provided by the referees, we have no choice but to return this manuscript with the message that we cannot offer to publish it.

Nevertheless, since the referees did have positive words for the goals of this study, we would not be opposed to considering a revised and extended manuscript based on this work. Any new submission would need to include new experimental data and further analyses thoroughly addressing the concerns raised by the reviewers. Without repeating all the points listed below, among the most fundamental issues are the following:
- The reviewers point out that the causal link between the SNPs and the observed variations in insulin response remains unclear, while alternative explanations need to be excluded. Importantly, reviewer #3 is concerned about the non-physiological doses of insulin used in the experiments, which could significantly affect the measured response.
- As reviewer #2 indicates, a truly integrative analysis providing conclusive mechanistic links between genetic variation, signaling response and insulin-responsive gene expression is at present lacking.

A resubmitted work would have a new number and receipt date. We recognize that this would

© European Molecular Biology Organization

involve substantial additional experimentation and analyses with unclear outcome and, as you probably understand, we can give no guarantee about its eventual acceptability. If you do decide to follow this course then it would be helpful to enclose with your re-submission an account of how the work has been altered in response to the points raised in the present review.

I am sorry that the review of your work did not result in a more favorable outcome on this occasion, but I hope that you will not be discouraged from sending your work to Molecular Systems Biology in the future. In any case, thank you for the opportunity to examine this work.

Reviewer #1:

The authors present a detailed study of the effects of insulin treatment on human skin fibroblasts in 35 individuals. The study integrates key insulin-sensitive signaling pathways along with genetic, gene expression and proteomic data. A key finding from the study is the significant variation in the phosphorylation of signaling proteins after insulin treatment. The authors integrate global gene expression data and identify many known insulin sensitive genes as well as genes that have not been described before as insulin sensitive. Many of the genes induced by insulin show time-specific as well as genetic dependent changes in gene expression. In addition to gene expression data the authors integrate proteomic data and identify many proteins that are repressed and induced during insulin treatment. Genomic analysis of cis regions indicated that many of the large variations in phosphorylation across individuals is a result of cis variation. The authors also identify significant trans regulation of specific downstream genes within STAT3 and p70S6K signaling pathways. Finally, the authors test the role of ERK in mediating gene expression changes and use both a chemical inhibitor and siRNA approach to show that ERK signaling influences insulin stimulated gene expression changes and proliferation. Overall, the study is well constructed and presented and is a wonderful example of integrative genetics approach to a specific biological pathway. I have a few specific critiques that should be addressed to make the manuscript easier for a broad audience to interpret.

Specific Comments
1. The cis parameters should be discussed in the text instead of the methods, as many readers will not be familiar with them
2. When discussing cis and trans results in the text it would be helpful to the reader to include the respective chromosomes next to the rsID.
3. Figure 5D. It would be helpful to label this figure with a legend in the corner.
4. Figure 6B. This figure is a bit confusing and does not appear to be well described in the methods or figure legend. It would helpful if the authors would expand on this.
5. The SILAC proteomic data is quite interesting. One of the proteins most repressed in response to insulin treatment is APOC3. I am not familiar with APOC3 expression in the fibroblasts. Since APOC3 influences insulin resistance and metabolic syndrome traits in mice, the authors might comment on this.

Reviewer #2:

In this manuscript, Wang and Cheung study the inter-individual variation of human fibroblasts to insulin stimulation. Using the Luminex platform, they observe substantial variation in the overall phosphorylation of multiple proteins downstream of the insulin receptor, including MEK-ERK, p70S6K, and STAT3. These individual-to-individual variations are coupled to insulin-stimulated transcriptional profiles, SILAC proteomic profiles, and SNP genotypes collected from the same cells. The paired profiles allow the authors to correlate datasets with one another to suggest hypotheses about the underlying variation. Wang and Cheung identify multiple SNP alleles that correlate with overall protein phosphorylation as well as gene expression, prompting the authors to propose that genomic differences are a major cause of the inter-individual variation.
There is the potential for this to be a compelling study, but a lot more work needs to be done to fill gaping holes and alternative explanations of the authors' finding. There are genetic differences, changes in overall phosphorylation states, and changes in gene expression, but the manuscript in its current form does not pursue any mechanisms for how these three datasets could be linked. Several

straightforward explanations (detailed below) are left unexplored, which leaves the manuscript feeling very descriptive.

Major points
1. The variation in responses across individuals could be related to covariates other than the SNPs the authors implicate. For example, there are three samples from infants... do these samples have disproportionately high or low levels of signaling for some of the phospho-proteins? How much variability can be attributed to variations in the cell preparation (as opposed to the cells themselves)? The authors say that the inter-individual variation of the phospho-proteins exceeded the technical duplicates, but how reproducible were the technical duplicates (this could be easily shown in the supplement as a scatter plot)? These data would be solidified with a second method (immunoblotting) to ensure that the fluctuations are not a strange artifact of the Luminex platform.
2. The observation that phospho-ERK, MEK, etc. varies among fibroblast preparations cannot be fully interpreted unless the total levels of the indicated proteins are similarly compared. If the total levels of the protein co-fluctuate with the magnitude of the phosphorylation events, then there is no difference in insulin-induced signal activation the results can be explained by protein expression levels. This is a critical set of data to obtain.
3. The STAT3 and p70S6K SNPs highlighted in Fig. 5C and 6D are both intronic SNPs that do not change the coding sequence of the protein. Thus, it is difficult to envision how these SNPs would influence the phosphorylation of those proteins. By contrast, intronic SNPs could influence splicing efficiency and propagate to total protein levels, which is why an assessment of total proteins (see Point #2 above) is so crucial.
4. The suitability of the manuscript for MSB would increase greatly if the authors could tie their observations into a pre-existing computational framework (e.g., PMID: 19357636). If inter-individual variations in the absolute levels in signaling proteins could give rise to the observed changes in phosphorylation, it would give a very satisfying explanation for the observed phenomena.

Minor points
1. The IP-immunoblots of Fig. 1 are lacking a positive control for IGF1R phosphorylation (e.g., IGF1 treatment). In the existing subpanel, there is a weak pTyr band in the plus insulin condition, but it is impossible to determine if it is truly weak or just an artifact of the chosen exposure.
2. Looking at Fig. 5A, one wonders if the individuals with the highest baseline phospho-protein are the ones with the highest insulin-induced phospho-protein, which would again point to similarities in signal activation. Indeed, fold activation has been shown to be a much more robust and informative metric of signal propagation (PMID: 20005851, 18957207, 20005850, 24530305). Fold activations shown in Fig. 2A are not related back to the basal activity states on a per-individual basis.
3. In Fig. 7A, there is no discussion of the 5-8 highest pERK individuals, who appear to have a completely different expression profile from the remaining individuals. Is this a thresholded response or is something else going on?
4. The introduction of the manuscript is not really appropriate for this journal. The inter-individual variations would be much more compelling if they related to total protein levels through SNPs, which then shifts the insulin signaling network toward a more- or less-responsive state to ligands. If this turns out to be true, then this becomes a very interesting systems-level consideration that should be set up properly in the introduction.

Technical points
1. The x-axis labels do not line up in Fig. 6A.
2. There are several typos in the manuscript: "within10", "the15" on page 10, "signal[ing] transduction" on page 12, and "insulin-induceD Protein Change' on the x axis of Fig. 4B.
3. Fig. 5D lacks a figure legend.
4. Fig. 6B is a low quality image and none of the gene names could be read. Overall this panel does not convey much information.

Reviewer #3:

Summary:
It appeared the authors' goal was to identify novel genes regulated by insulin using an approach that combines genomic data, gene expression analysis, proteomic analysis, and monitoring the signaling state of multiple pathways simultaneously. To this end, 35 lines of primary human foreskin fibroblasts were stimulated with high-dose insulin at different times to acquire the data. More than 20% of expressed genes were found to be altered in response to insulin, as gauged by microarray. The authors noticed large variations in the fold response of cell lines from different donors and proceeded to stratify the data by donor line and calculated correlations between signaling pathway induction, gene expression, phosphoproteomics, and SNPs. Finally, the authors investigate a few genes and their dependence on ERK signaling.

General Remarks:
The authors' conclusion that individual variability in cellular responses to insulin is supported, but it is not surprising. Given that normal human blood insulin levels seldom exceed 1 or 2nM tops (Dalla Man C et al. Diabetes 2005;54:3265-3273. and Daly ME, et al. Am J Clin Nutr 1998;67:1186-96.), it is hard to gauge the physiological relevance of 100nM insulin stimulation on skin cells. It seems possible that some of the variation in cell responses may come from the high dose of insulin. Under the short 5-10 min stimulations perhaps the high dose is more interpretable, but 6 hours of 100nM insulin is certainly beyond what any human cell might ever see. Novelty is an issue with some of the data. For example, the final panel, Figure 7E, which shows that ERK knockdown blocks proliferation, has been known for a long time (reviewed in Chambard JC, et al. BBA 2007;1773:1299-1310).

That aside, the power of this study lies in the combination of these techniques to get a global picture of signaling responses that can be cross-referenced with the genotypes of the given cells. The presented results could be more useful if placed in an accessible format as a searchable resource for other investigators.
Diabetes and cell signaling researchers may find this research interesting.

Major Comments:
1. What line was the first figure derived from, given that the 35 different lines have variable responses to insulin and might generate different dose-response curves?

2. How was the dose of insulin for stimulation chosen? 100nM is at least 10- to 100-times greater than insulin levels in human blood. The authors should discuss the supraphysiological dose and any potential impact it might have on interpreting the data.

3. It should be noted in the discussion that the cells came from newborns, and that cell responses in adults might be different.
a. Have any studies correlated age and insulin signaling in any other primary tissues?

4. The authors should provide full a list of results in excel spreadsheet or csv format for the microarray data, analyzed LC/MS-MS data, and the Luminex data. The PDF is not user-friendly.
a. Table E2 is missing from the supplemental info. PDF has pages 1-46 but not page 47.

5. Figure legends need more description.

6. Are the data in 5A the same as in 2B? If so, it should be clearly pointed out that the same data have been replotted to show non-normalized values and that these data are not from a separate experiment.

7. Are the SNPs that affect kinase activity in the protein coding regions and do they change the coded residue? Further discussion of how these SNPs might be impacting gene function would be helpful.

8. How is gene ontology informative when 30% of all expressed genes are significantly changed?

9. In the microarray data analysis, was false discovery rate calculated? The Affy TAC software gives FDR as well as ANOVA p-value and the data should stratified by the FDR. This might reduce the apparent number of changed genes significantly.
a. 2637 changed genes / 11845 expressed genes is 22% and not 30%.

10. Panels like figure 5B should be generated for each phosphoprotein readout. Especially for ERK, because that is a main focus.

11. On page 10, please give further explanation of what this means and how it was determined: "ERK activation explains at least 16% and as much as 44% of individual variability in insulin-induced changes in expression of these genes"

12. In Fig 7D, for ENC1, 3 outlier points seem to account for the correlation, without those 3 on the upper right what would the correlation be?
a. Also, in the experiment with U0126, an important control is U0126 alone. This will show whether ENC1 and GEM increase in response to insulin in the presence of MEK inhibition.
b. ENC1 and GEM are not discussed at all until figure 7D. How were they chosen?

Minor comments:
1. In figure 2B, the mean should be shown on the fold changes.
2. In the results for Fig 4, it should be pointed out that the data are averages.
3. Figure 6B is not helpful because genes connected to the kinases cannot be seen. Perhaps this could be shown in a higher resolution or a table of some kind.

New Submission 11 May 2015

(please see next page)

We thank the reviewers for the detailed suggestions and comments. The suggested experiments and analyses have strengthened our conclusions. We appreciate the insights and thoughtful comments of the reviewers.

Reviewer #1:
Specific Comments
1. The cis parameters should be discussed in the text instead of the methods, as many readers will not be familiar with them

We have included the cis parameters into the main text (Pg 9). The text reads:

"To narrow the search, we tested for allelic associations between DNA sequence variants within the genes that encode the signaling proteins and the extent of insulin-induced phosphorylation. SNPs within the 7 signaling proteins (10 kb upstream of 5' UTR to 10 kb downstream of 3' UTR; minor allele frequency >5%) were selected from dbSNP (Smigielski et al, 2000) and genotyped in our subjects. By association analysis, we found that the activation of several signaling proteins was in part cis-regulated (Fig 5B; 5C)."

2. When discussing cis and trans results in the text it would be helpful to the reader to include the respective chromosomes next to the rsID.

We have now included the chromosome number and nucleotide position of each SNP next to the rsIDs in main text.

3. Figure 5D. It would be helpful to label this figure with a legend in the corner.

Legend has been added in the corner of the figure (now 5C). Thank you for pointing it out.

4. Figure 6B. This figure is a bit confusing and does not appear to be well described in the methods or figure legend. It would helpful if the authors would expand on this.

We have removed Figure 6B to make the manuscript more concise and focused. In addition we included the list of phosphorylation target – gene pairs that are correlated (r>0.4) in Supplementary Table S1.

5. The SILAC proteomic data is quite interesting. One of the proteins most repressed in response to insulin treatment is APOC3. I am not familiar with APOC3 expression in the fibroblasts. Since APOC3 influences insulin resistance and metabolic syndrome traits in mice, the authors might comment on this.
Thank you for the suggestion. We have added a discussion of APOC3 (Pg 7). The text reads:

“These “insulin-responsive proteins” play a role in protein biosynthesis (EIF1, RPS6), RNA processing (RBM25, HNRNPC), lipid biosynthesis (APOC3, FASN) and protein transport (XPO5, TMED3). For instance, it was previously reported that abnormal level of APOC3 led to hypertriglyceridemia in transgenic mice and human (Carlson & Ballantyne, 1976; Ginsberg et al, 1986; Ito et al, 1990; Aalto-Setälä et al, 1992; Pollin et al, 2008). APOC3 is transcriptionally repressed by insulin, a critical step of its regulation (Chen et al, 1994; Li et al, 1995). Here we showed that APOC3 protein is down-regulated following insulin treatment, supporting its role in mediating insulin response.”

Reviewer #2:

Major points

1. The variation in responses across individuals could be related to covariates other than the SNPs the authors implicate. For example, there are three samples from infants... do these samples have disproportionately high or low levels of signaling for some of the phospho-proteins?

All the samples were foreskin-derived fibroblasts from newborn males; thus the usual covariates of gender and age have been controlled in our project.

How much variability can be attributed to variations in the cell preparation (as opposed to the cells themselves)? The authors say that the inter-individual variation of the phospho-proteins exceeded the technical duplicates, but how reproducible were the technical duplicates (this could be easily shown in the supplement as a scatter plot)? These data would be solidified with a second method (immunoblotting) to ensure that the fluctuations are not a strange artifact of the Luminex platform.

We were careful to minimize technical variability and to replicate the findings. All the samples were treated in same ways so cell preparation is unlikely to explain the variability in phosphorylation. The strongest evidence for biological basis of the individual differences is finding the genetic contributions since technical differences could not track with genotypes.

To address the reviewer's queries, we have included scatter plots showing that the Luminex results from duplicates are highly similar (Supplementary Fig S2). For the duplicates, cells from the same individuals were cultured, treated and assayed independently.

Supplemental Fig S2

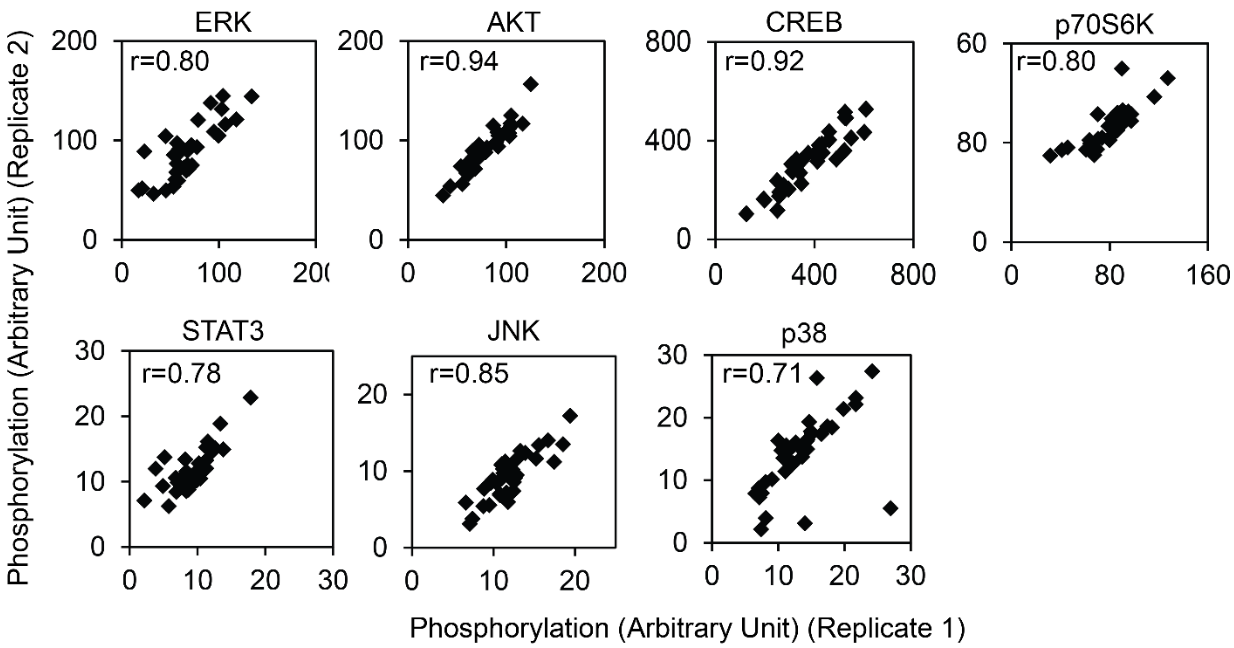

Supplementary Fig S2. Luminex assay results from biological duplicates are highly similar. Fibroblasts from 35 individuals were seeded in duplicate wells, serum-starved and treated with 100nM insulin for 10 minutes independently. Cells were lysed and phosphorylation of each target is measured by Luminex assay.

However, we see the value of using an independent method as suggested by the reviewer. So we carried out immunoblotting of samples from 3 individuals including those with the highest and lowest phosphorylation of ERK. We showed that the antibody is specific for its target, and immunoblotting replicates the results of Luminex assays while the latter has broader dynamic range (Supplementary Fig S3A).

Supplementary Fig S3

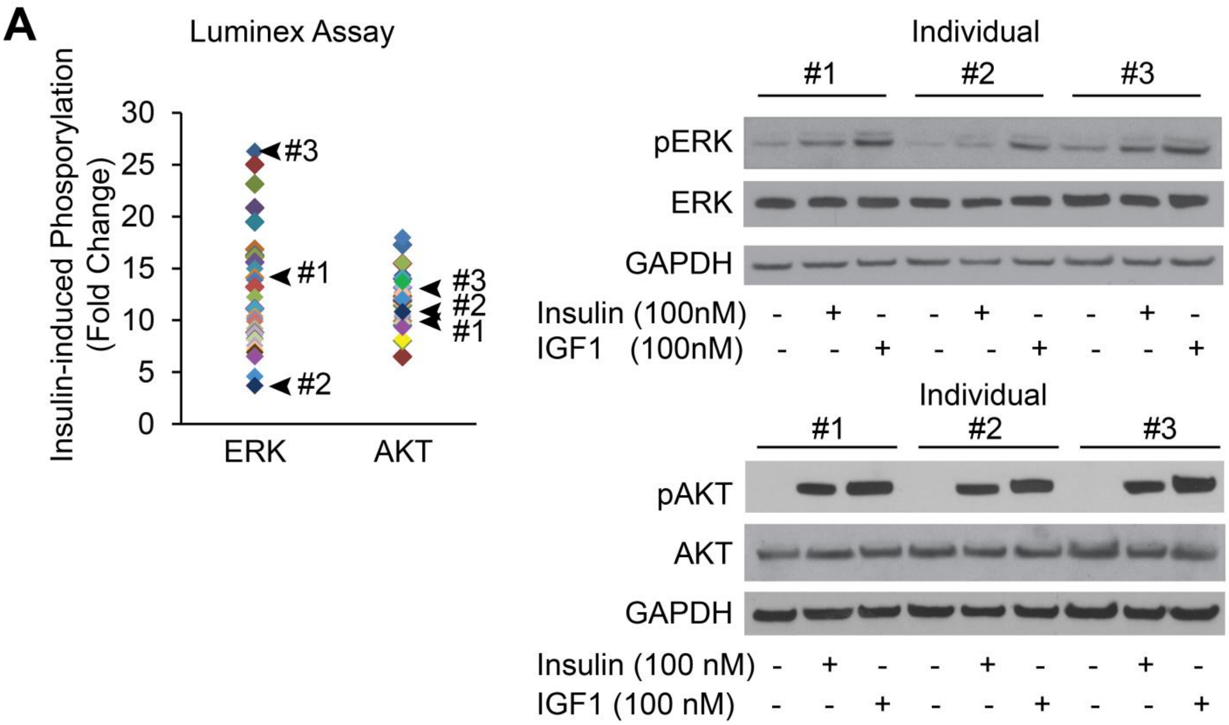

Supplementary Fig S3. Luminex phosphorylation measurements are reproducible and have broad dynamic range. A) Individual difference in insulin-induced phosphorylation of ERK measured by Luminex assay is validated by western blot. Skin fibroblasts from indicated 3 individuals were treated with 100nM insulin in independent duplicates. Cells were harvested and assayed using Luminex phosphorylation assay or western blot using the same antibodies. Cells were treated with 100 nM IGF1 as a positive control. Total protein levels of AKT or ERK did not change before and after insulin treatment.

2. The observation that phospho-ERK, MEK, etc. varies among fibroblast preparations cannot be fully interpreted unless the total levels of the indicated proteins are similarly compared. If the total levels of the protein co-fluctuate with the magnitude of the phosphorylation events, then

there is no difference in insulin-induced signal activation the results can be explained by protein expression levels. This is a critical set of data to obtain.

We appreciate the reviewer's suggestion. We have now included data showing that total protein levels do not differ across individuals and therefore do not contribute to the individual variation in phosphorylation (Supplementary Fig S3B).

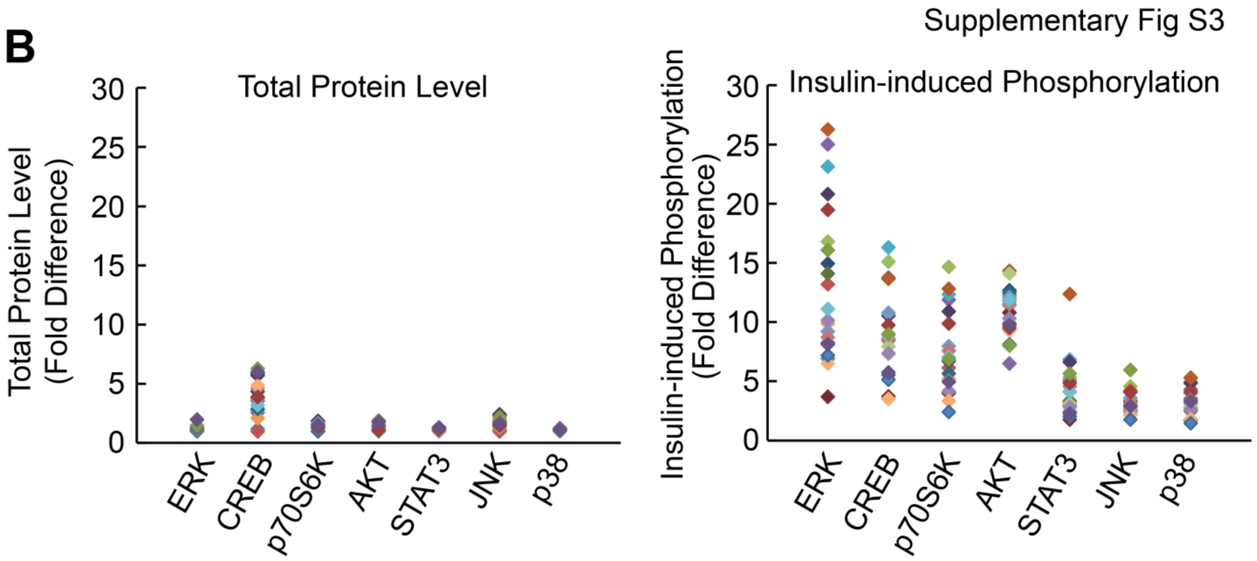

Supplementary Fig S3 B) Compared to differences in phosphorylation levels, total protein levels are not as variable across individuals. Total protein levels or insulin-induced phosphorylation levels of 7 signaling factors shown in Figure 5A were measured in 20 individuals by Luminex assay. Average value of biological duplicates is shown.

Moreover, protein levels of signaling factors remain the same before and after insulin treatment (Supplementary Fig S3A, shown above). In addition, as we focus on response to insulin in this study, we calculated the fold change in phosphorylation before and after insulin treatment in the same individual, which is not affected by individual difference in total protein level.

The text (Pg 8) reads,

"Using western blot analysis, we confirmed the individual variation in insulin-induced phosphorylation of target protein such as AKT and ERK (Supplementary Fig S3). Moreover, the differences in phosphorylation of AKT and ERK are not due to differences in expression levels of total AKT and ERK since those do not vary among individuals, and showed little changes following insulin treatment (Supplementary Fig S3). These results uncover that upon insulin binding to its receptor, even in the early steps of activation of signal transduction pathway, there are significant individual differences in response to insulin."

3. The STAT3 and p70S6K SNPs highlighted in Fig. 5C and 6D are both intronic SNPs that do not change the coding sequence of the protein. Thus, it is difficult to envision how these SNPs would influence the phosphorylation of those proteins. By contrast, intronic SNPs could

influence splicing efficiency and propagate to total protein levels, which is why an assessment of total proteins (see Point #2 above) is so crucial.

As noted above, we have added data to show that the total protein levels do not vary between individuals. We believe these intronic SNPs are not causal variants, but rather tagging those functional variants.

4. The suitability of the manuscript for MSB would increase greatly if the authors could tie their observations into a pre-existing computational framework (e.g., PMID: 19357636). If inter-individual variations in the absolute levels in signaling proteins could give rise to the observed changes in phosphorylation, it would give a very satisfying explanation for the observed phenomena.

Following reviewer's suggestion, we referred to the previous study showing crosstalk between pathways downstream to EGF receptor and insulin receptor, and asked whether such crosstalk can be detected in our subjects. We found that there are some suggestions for allelic association of SNPs in EGFR with phosphorylation of ERK and AKT (nominal P<0.03; see figure below).

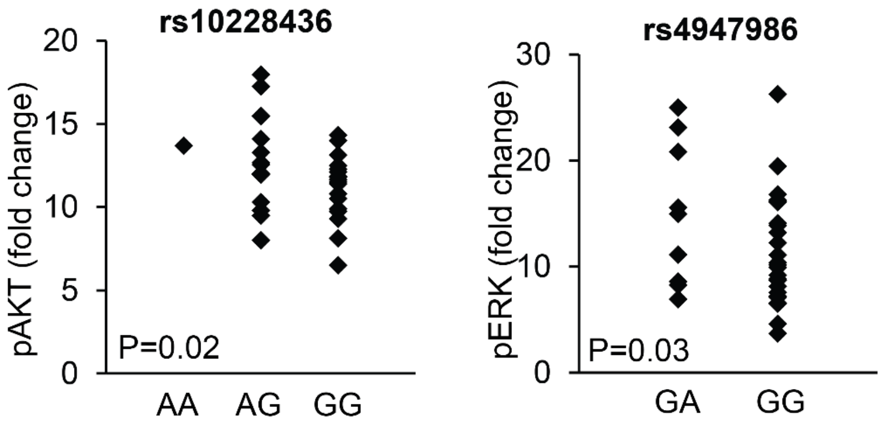

We describe results of this analysis in the main text, which reads:

"Previous studies have shown that cells respond to multiple extracellular stimuli through crosstalks between different pathways. For instance, the EGF- and insulin-induced signaling pathways share some downstream factors and insulin treatment enhances ERK phosphorylation stimulated by EGF (Borisov et al, 2009). We therefore asked whether variants in EGF receptor gene (EGFR) contribute to different levels of insulin-induced phosphorylation. We obtained genotype of 17 SNPs within EGFR (minor allele frequency >5%), and analyzed the association between these variants and phosphorylation of ERK and AKT. Individuals carrying A allele at rs10228436 (chr7:55170575) show higher phosphorylation of AKT than those carrying G allele (nominal P-value = 0.02). Similarly variants at rs4947986 (chr7:55153962) are associated with ERK phosphorylation (nominal P-value = 0.03). However, these associations are weak as none of them is significant after correction for multiple testing. Given the crosstalks

between pathways, it is likely that several regulators in addition to EGF receptor affect ERK and AKT phosphorylation and a larger sample is needed to identify complex genetic interactions."

Minor points
1. The IP-immunoblots of Fig. 1 are lacking a positive control for IGF1R phosphorylation (e.g., IGF1 treatment). In the existing subpanel, there is a weak pTyr band in the plus insulin condition, but it is impossible to determine if it is truly weak or just an artifact of the chosen exposure.

We have treated the cells with IGF1 as positive control (Supplementary Fig S1A). As expected, IGF1 activates IGF1R, and downstream signaling factors including ERK and AKT (Supplementary Fig S1A, S3B). For pTyr band, we have repeated the same experiments in multiple individuals; the very faint band is not present in all samples and is likely a non-specific band.

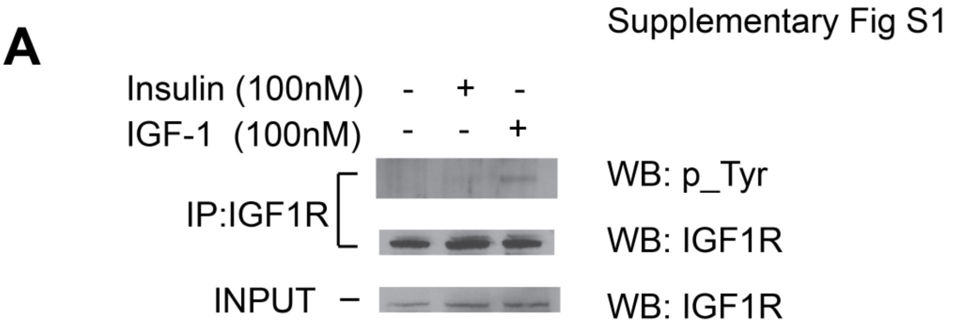

Supplementary Fig S1. (A) IGF1R is activated by IGF-1, shown as control experiment for Fig 1A. Fibroblasts were treated with IGF-1 or Insulin before IGF1R was immunoprecipitated and analyzed for phosphorylation of tyrosine.

Supplementary Fig S3

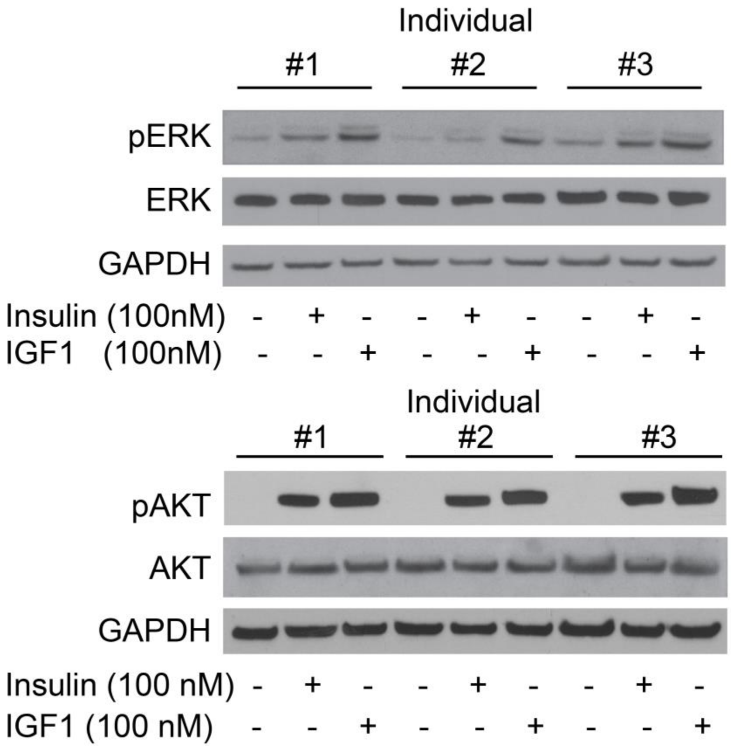

Supplementary Fig S3. Luminex phosphorylation measurements are reproducible and have broad dynamic range. (A) Individual difference in insulin-induced phosphorylation of ERK measured by Luminex assay is validated by western blot. Skin fibroblasts from indicated 3 individuals were treated with 100nM insulin in independent duplicates. Cells were harvested and assayed using Luminex phosphorylation assay or western blot using the same antibodies. Cells were treated with 100 nM IGF1 as a positive control. Total protein levels of AKT or ERK did not change before and after insulin treatment.

2. Looking at Fig. 5A, one wonders if the individuals with the highest baseline phospho-protein are the ones with the highest insulin-induced phospho-protein, which would again point to similarities in signal activation. Indeed, fold activation has been shown to be a much more robust and informative metric of signal propagation (PMID: 20005851, 18957207, 20005850, 24530305). Fold activations shown in Fig. 2A are not related back to the basal activity states on a per-individual basis.

We have included basal activity of each individual and show that the individuals with the highest baseline phospho-protein do not correspond exactly to the individuals with the highest insulin-induced phospho-protein (Supplementary Fig S4; shown below). Moreover, individual variation in insulin-induced phosphorylation of signaling proteins is greater than that at baseline. We

agree with the reviewer, the fold activation therefore is more informative, and therefore we focus on fold activation in this study.

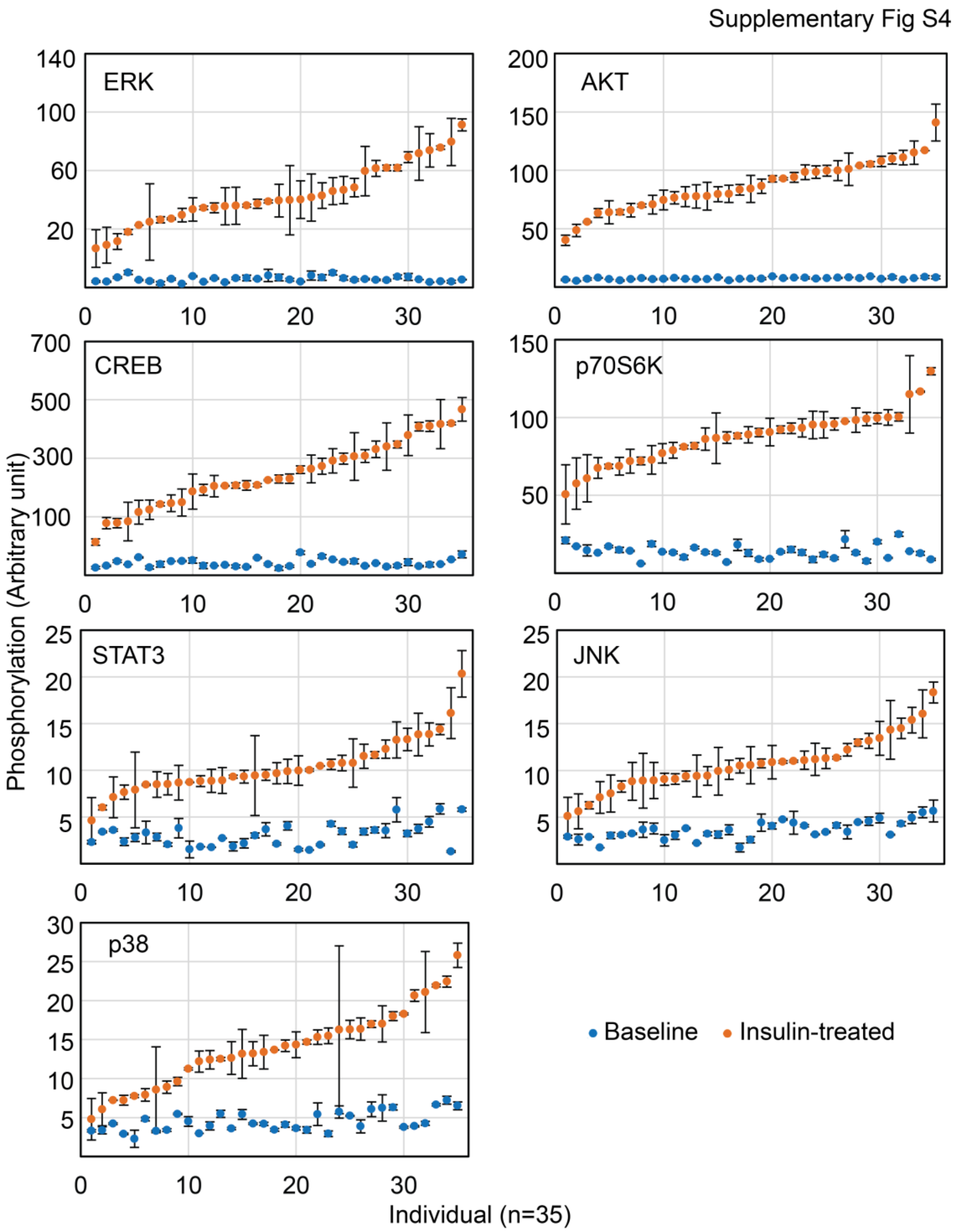

Supplementary Fig S4. Phosphorylation levels of signaling factors after insulin treatment do not show significant correlations with those at baseline. Phosphorylation of each signaling factor at baseline or after insulin treatment from the same individual is shown in the same column. Variation among individuals in insulin-induced phosphorylation is significantly greater than that within replicated assays ($P<0.05$; ANOVA) for all seven signaling factors. The average phosphorylation level of replicates is shown. Error bar = SEM of biological replicates.

3. In Fig. 7A, there is no discussion of the 5-8 highest pERK individuals, who appear to have a completely different expression profile from the remaining individuals. Is this a thresholded response or is something else going on?

It is suggestive of a threshold effect – only genes that are correlated with phosphorylation of signaling factor (r>0.4) are shown in the heatmap.

4. The introduction of the manuscript is not really appropriate for this journal.

We have rewritten the Introduction to give a broader overview while specifying more clearly the goals of our study. We hope the reviewer agrees that the revised text is more informative.

The inter-individual variations would be much more compelling if they related to total protein levels through SNPs, which then shifts the insulin signaling network toward a more- or less-responsive state to ligands. If this turns out to be true, then this becomes a very interesting systems-level consideration that should be set up properly in the introduction.

As shown above (in our reply to query #2), there is little individual differences in total protein levels, and the variation is in the activation of the phospho-proteins.

Technical points
1. The x-axis labels do not line up in Fig. 6A.

We have aligned the label. Thank for your pointing it out.

2. There are several typos in the manuscript: "within10", "the15" on page 10, "signal[ing] transduction" on page 12, and "insulin-induceD Protein Change' on the x axis of Fig. 4B.

We apologize for the typos. We corrected the above and read the manuscript more carefully.

3. Fig. 5D lacks a figure legend.

We have added the legend; we apologized for the omission. We thank the reviewer for noticing it.

4. Fig. 6B is a low quality image and none of the gene names could be read. Overall this panel does not convey much information.

We agree with the reviewer and have deleted this figure.

Reviewer #3:

Major Comments:
1. What line was the first figure derived from, given that the 35 different lines have variable responses to insulin and might generate different dose-response curves?

The sample shown in Fig 1 is the individual ranked 16th in AKT phosphorylation induced by insulin. To address reviewer's question, we treated cells from two additional individuals with a serial titration of insulin (shown below; Supplementary Fig S1). Similar trends are seen in all three individuals.

Supplementary Fig S1

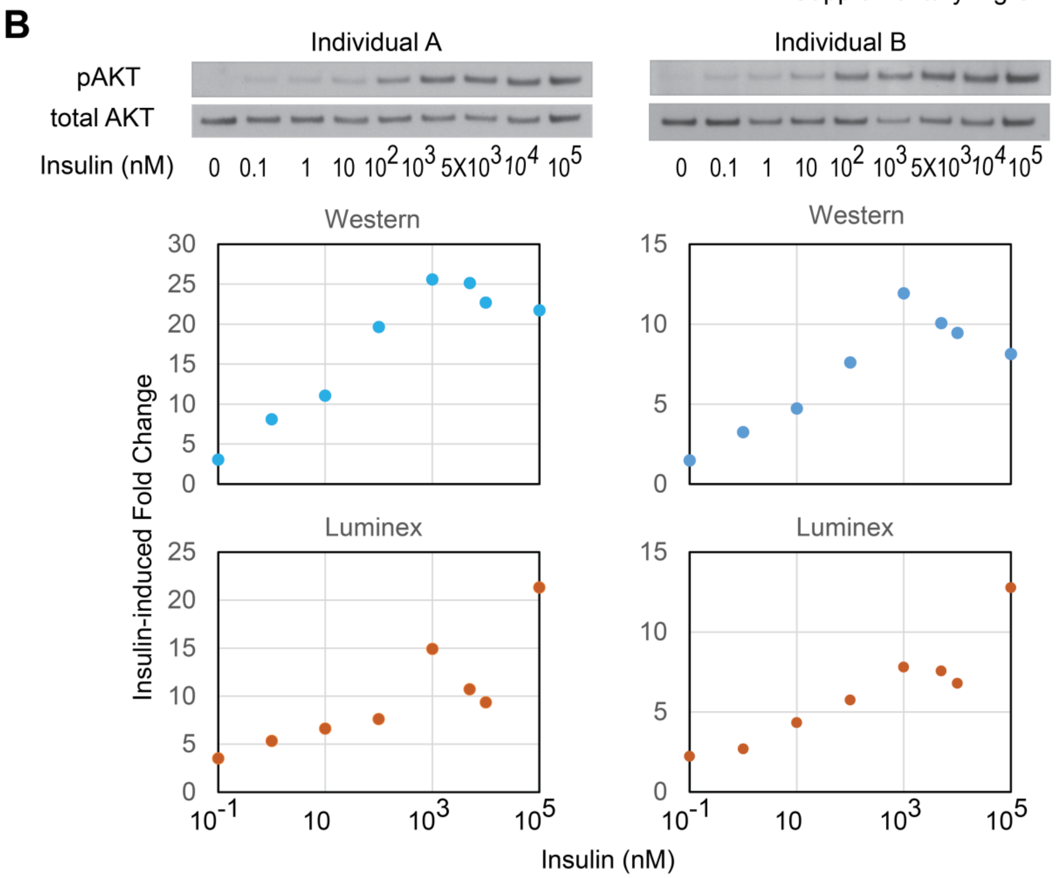

Supplementary Fig S1. (B) Dose-dependent insulin response in skin fibroblasts. Skin fibroblasts from two individuals were treated with a serial dilution of insulin (0.1-10^5 nM) for 10 minutes. Whole cell lysates were harvested and analyzed by western blot and Luminex phosphorylation assay in parallel using antibodies against phospho-Ser473 of AKT or total AKT. Western blot signal was quantified using Image J. Fold change of AKT phosphorylation following insulin treatment is shown.

2. How was the dose of insulin for stimulation chosen? 100nM is at least 10- to 100-times greater than insulin levels in human blood. The authors should discuss the supraphysiological dose and any potential impact it might have on interpreting the data.

We were aware of the difference between 100nM of serum insulin level *in vivo*. In order to find the optimal dosage for cell culture, we tested for responses at different insulin dose, and 100 nM is in the middle of dynamic range (figure above; Supplementary Fig S1). The response seems to saturate at 1μM insulin. This is also consistent with concentration used in fibroblasts in studies by other groups.

We thank the reviewer for this question. We have now added our reply to the main text (Pg 5) which reads,

"To determine the optimal dose of insulin, we treated cells with serial titration of insulin and chose the concentration in the midpoint of dose-response curve (100nM), this is the same dose used for cultured fibroblast in other studies (Frittitta et al, 1998; Borisov et al, 2009). Under our experimental conditions, insulin activates the insulin receptor in fibroblasts but not the insulin-like growth factor 1 receptor (Fig 1A; Supplementary Fig S1)."

3. It should be noted in the discussion that the cells came from newborns, and that cell responses in adults might be different.

Indeed seeing these results in adults will allow us to understand the genetics findings in different context; one could study the influence of different environments on the genetics. We have added this to the discussion (Pg 13), which reads

"*Our study was carried out using samples from newborns. It would be interesting to carry out similar analyses in adults which would provide additional information on how environmental factors, such as adult diets, influence the genetic effects.*"

a. Have any studies correlated age and insulin signaling in any other primary tissues?
We believe the genetic factors we identified here that affect insulin response in newborn fibroblasts are likely to contribute to insulin response in other tissues and in older individuals. To understand further how these same genetic factors play a role in adults is beyond scope of this study.

4. The authors should provide full a list of results in excel spreadsheet or csv format for the microarray data, analyzed LC/MS-MS data, and the Luminex data. The PDF is not user-friendly.

Raw microarray data have been deposited in GEO. All related raw data, including gene expression, LC/MS-MS and Luminex data have been submitted in csv format under the same GEO accession number (GSE21891). Reviewer access link:
http://www.ncbi.nlm.nih.gov/geo/query/acc.cgi?token=tdavtqiaqcsycxu&acc=GSE21891

a. Table E2 is missing from the supplemental info. PDF has pages 1-46 but not page 47.

We apologize for the omission; we have added the information. Thank you for pointing it out.

5. Figure legends need more description.
We added more details to the figure legends.

6. Are the data in 5A the same as in 2B? If so, it should be clearly pointed out that the same data have been replotted to show non-normalized values and that these data are not from a separate experiment.
They are the same data. We have now clarified it; the legend for 5A (Pg 27) now reads,

"*Phosphorylation of signaling factors differs extensively among individuals following insulin treatment compared to those at baseline. The data in Figure 2A are re-plotted here to contrast the phosphorylation before and after insulin treatment.*"

7. Are the SNPs that affect kinase activity in the protein coding regions and do they change the coded residue? Further discussion of how these SNPs might be impacting gene function would be helpful.

The SNPs are intronic; however, as in all association study, we do not claim that the test SNPs are causal, mostly likely they tag functional variants.

8. How is gene ontology informative when 30% of all expressed genes are significantly changed?

We provided the ontology information to allow the genes to be grouped. The number of significant genes does not diminish the need to annotate them; we think it may even make it more important to categorize them. We thought it will be easier for the readers to go through the list of genes if given some categories rather than having to read a long list of genes.

9. In the microarray data analysis, was false discovery rate calculated? The Affy TAC software gives FDR as well as ANOVA p-value and the data should stratified by the FDR. This might reduce the apparent number of changed genes significantly.

The P-value threshold 10^{-6} we applied to ANOVA analysis has taken into account effect of testing ~11,800 genes. Following reviewer's suggestion, we calculated FDR. At FDR 0.05, expression of 35% genes significantly changed following insulin treatment, and at FDR 0.01, 26% genes are significant.

a. 2637 changed genes / 11845 expressed genes is 22% and not 30%.
Thank you for pointing it out. We have made the correction (Pg 6).

10. Panels like figure 5B should be generated for each phosphoprotein readout. Especially for ERK, because that is a main focus.

We have included results of all signaling factors (shown below; Supplementary Fig S4). They all showed extensive variation at phosphorylation after insulin treatment.

Supplementary Fig S4

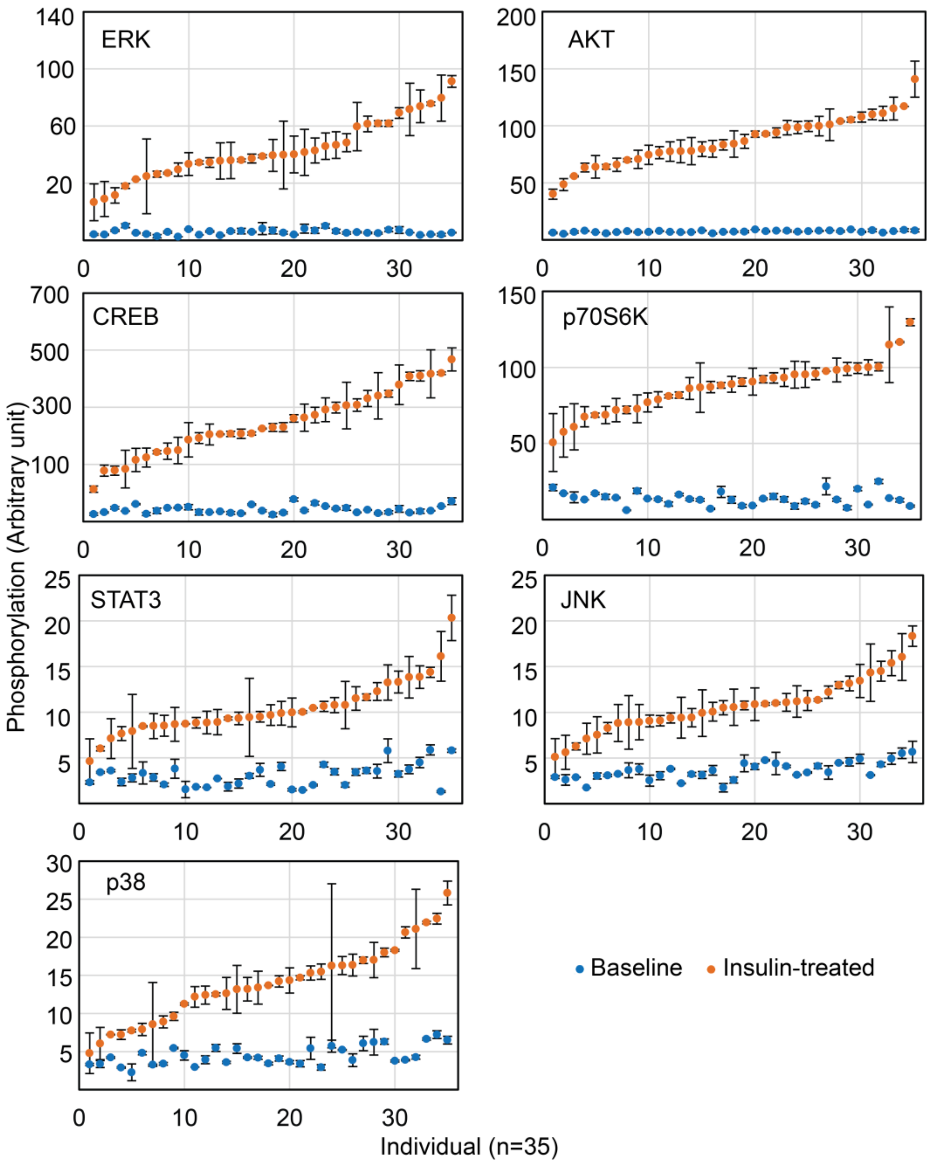

Supplementary Fig S4. Phosphorylation levels of signaling factors after insulin treatment do not show significant correlations with those at baseline. Phosphorylation of each signaling factor at baseline or after insulin treatment from the same individual is shown in the same column. Variation among individuals in insulin-induced phosphorylation is significantly greater than that within replicated assays ($P<0.05$; ANOVA) for all seven signaling factors. The average phosphorylation level of replicates is shown. Error bar = SEM of biological replicates.

11. On page 10, please give further explanation of what this means and how it was determined: "ERK activation explains at least 16% and as much as 44% of individual variability in insulin-induced changes in expression of these genes

We calculated the R^2 as the proportion of variance explained as in standard correlation analysis. The correlation between gene expression and protein phosphorylation allows us to determine the proportion of variability in gene expression explained by ERK activation.

12. In Fig 7D, for ENC1, 3 outlier points seem to account for the correlation, without those 3 on the upper right what would the correlation be?

The analysis is based on 35 samples. Removing any 3 data points certainly will affect the correlation. We noted that correlation alone does not mean causal relationships. Therefore we inhibited the kinase pharmacologically and studied the effect. Our results showed that inhibition of pERK led to less induction of ENC1 gene expression, providing experimental evidence that ERK phosphorylation regulates *ENC1* expression in response to insulin.

a. Also, in the experiment with U0126, an important control is U0126 alone. This will show whether ENC1 and GEM increase in response to insulin in the presence of MEK inhibition.
b. ENC1 and GEM are not discussed at all until figure 7D. How were they chosen?

In this experiment, we serum starved the cells to set the baseline, followed by treatment with DMSO as negative control, DMSO with insulin, and U0126 with insulin. UO126 alone following serum starvation was not performed. We reasoned that U0126 treatment in absence of all growth factors is unlikely to generate meaningful result.

ENC1 and *GEM* are two examples that appear interesting to us for follow up studies.

Minor comments:
1. In figure 2B, the mean should be shown on the fold changes.

In Figure 2B, each dot represents the mean fold change of replicates from the same individual. We did not include the error bars in this figure since they are hard to see (as illustrated in the figure below). The same data were re-plotted in Supplementary Fig S4 (shown above) in order to show mean and SEM of each individual before and after insulin treatment.

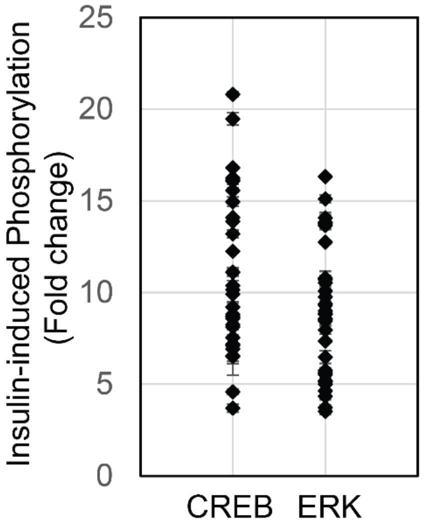

2. In the results for Fig 4, it should be pointed out that the data are averages.

We have added this to the legend (Pg 27) which reads:

"Correlations of protein and gene expression levels before and after insulin treatment are shown. The gene expression levels are averages from our 35 subjects.."

3. Figure 6B is not helpful because genes connected to the kinases cannot be seen. Perhaps this could be shown in a higher resolution or a table of some kind.

We agree with the reviewer and have removed Figure 6B.

2nd Editorial Decision 11 May 2015

Thank you again for submitting your work to Molecular Systems Biology. We sent your manuscript to the previous reviewers #2 and #3 (current reviewer #1). We have now heard back from these two referees who think that the study has been improved. However, reviewer #2 raises two remaining concerns on your work, which should be convincingly addressed in a revision of the manuscript. As you will see below, the recommendations of reviewer #2 regarding these remaining issues are clear so there is no need to repeat them here.

If you feel you can satisfactorily deal with these points and those listed by the referees, you may wish to submit a revised version of your manuscript. Please attach a covering letter giving details of the way in which you have handled each of the points raised by the referees. A revised manuscript will be once again subject to review and you probably understand that we can give you no guarantee at this stage that the eventual outcome will be favorable.

Reviewer #1:

The authors addressed most of my concerns, although it should still be stated a little more bluntly in the text what the physiological range of insulin concentrations are compared to what had to be used in vitro with the foreskin fibroblasts.

Also, the hits that were focused on, ENC1 and GEM, should be discussed in more detail. Maybe some info about what they do and how come they might be involved in the dynamic response to insulin across independent biological samples?

Reviewer #2:

The authors have improved their manuscript in various aspects since the first submission. However two core issues, raised in the first review, still have not been satisfactorily addressed.

Major points
1. The authors should be commended for supplementing their phospho data with data on total protein. However, the display shown on Fig. S3B is not the comparison of interest. Clearly, the fold change of total proteins is not expected to change greatly upon acute insulin stimulation. The key comparison is whether the unstimulated levels of total protein ACROSS SAMPLES correlates with the extent of insulin stimulated phosphorylation. For example, one could envision a scenario where the ERK pathway is always stoichiometrically phosphorylated upon insulin stimulation; thus, the variation in observed ERK phosphorylation could be completely explained by the sample-to-sample variation in total ERK protein. The authors can readily assess this with their existing data by plotting fold-changes in phosphorylation vs. unstimulated Luminex fluorescence for the corresponding total protein.
2. The authors make a big point to say that SNPs give rise to cis changes in phosphorylation. However, as multiple reviewers pointed out in the first review, the polymorphisms shown are all intronic, and therefore they are unlikely to be the causal variant. Therefore, the authors have not definitely shown a cis effect, because the observed association could simply be a chance correlation (even after multiple-hypothesis correction). From the perspective of protein phosphorylation, the two ways that a SNP can act in cis are by changing the abundance of the molecule (data that the authors already have in hand) or by changing the coding sequence to alter docking sequences, localization, etc. This could be tested by sequencing the coding region of the proteins of interest in the samples (or, in at least a handful of samples). Otherwise, the entire basis of the cis finding is phenomenological.

Minor points

1. The addition of IGF1 stimulation as a positive control in Fig. S1A is welcome, but that positive control is so important that this experiment should replace the IP in Fig. 1A.
2. The total ERK and phospho-AKT blots on Fig. S3A look burned in, which will obscure possible quantitative differences among these lanes (PMID: 25852189). A lighter exposure should be shown.
3. The rightmost y-axis labels on Fig. S2 is cut off.
4. Fig. 7A is missing a color bar to depict the range of values.
5. I missed this in the first review, but the expression of TIGAR and PER2 are not highlighted in Fig. 7A for high correlation with insulin-induced ERK phosphorylation. The authors should explain why the expression of TIGAR and PER2 are selected to validate the ERK-dependent gene expression in Fig. 7B.
6. Gene symbols should be consistent in the manuscript. For example, the authors use TIGAR in Fig 7B, but C12orf5 in Supplementary Table S1.
7. Page 3 should read "food intake".

1st Revision - authors' response 27 May 2015

We thank the reviewers for their comments and suggestions in this and previous reviews. We appreciate their help in improving our manuscript.

Reviewer #1:

The authors addressed most of my concerns, although it should still be stated a little more bluntly in the text what the physiological range of insulin concentrations are compared to what had to be used in vitro with the foreskin fibroblasts.

We follow the reviewer's suggestion and explicitly stated in the text the physiological insulin levels in human plasma (0.1~2 nM) (Page 5). The text reads:
"Human physiological plasma insulin levels are about 0.1~2 nM (Melmed et al, 2011); however, we expected a different concentration of insulin would be needed in cell-based experiments. To determine the optimal dose of insulin, we treated cells with serial titration of insulin and chose the concentration in the midpoint of dose-response curve (100nM), which is the same dose used for cultured fibroblast in other studies (Frittitta et al, 1998; Borisov et al, 2009)."

Also, the hits that were focused on, ENC1 and GEM, should be discussed in more detail. Maybe some info about what they do and how come they might be involved in the dynamic response to insulin across independent biological samples?

We discussed in detail the functions of ENC1 and GEM, and the implications of our findings (Page 11). The text reads:
"ENC1 encodes an actin-binding protein which is involved in adipocyte differentiation (Zhao et al, 2000). Its overexpression is also associated with various cancers (Fujita et al, 2001; Hammarsund et al, 2004; Durand et al, 2011). The positive correlation between ENC1 expression and ERK phosphorylation in response to insulin suggests a possible mechanism for higher cancer risk in patients with insulin resistance (Arcidiacono et al, 2012). GEM encodes an RGK family of GTPase, which regulates voltage-dependent Ca2+ channels. It has been shown that Gem-null mice were glucose intolerant and have impaired insulin secretion (Gunton et al, 2012). Here we showed that gene expression of GEM is regulated by insulin-stimulated ERK phosphorylation, suggesting a feedback pathway that between insulin secretion and insulin signaling."

Reviewer #2:

The authors have improved their manuscript in various aspects since the first submission. However two core issues, raised in the first review, still have not been satisfactorily addressed.

Major points
1. The authors should be commended for supplementing their phospho data with data on total protein. However, the display shown on Fig. S3B is not the comparison of interest. Clearly, the fold change of total proteins is not expected to change greatly upon acute insulin stimulation. The key

comparison is whether the unstimulated levels of total protein ACROSS SAMPLES correlates with the extent of insulin stimulated phosphorylation. For example, one could envision a scenario where the ERK pathway is always stoichiometrically phosphorylated upon insulin stimulation; thus, the variation in observed ERK phosphorylation could be completely explained by the sample-to-sample variation in total ERK protein. The authors can readily assess this with their existing data by plotting fold-changes in phosphorylation vs. unstimulated Luminex fluorescence for the corresponding total protein.
In Fig S3B, we compare unstimulated total protein levels (left panel) and insulin-stimulated phosphorylation of same proteins (right panel) across sample. We showed that these two are not correlated, and therefore the variation exists mostly at the insulin-stimulated phosphorylation level, which cannot be explained by total protein level.

2. The authors make a big point to say that SNPs give rise to cis changes in phosphorylation. However, as multiple reviewers pointed out in the first review, the polymorphisms shown are all intronic, and therefore they are unlikely to be the causal variant. Therefore, the authors have not definitely shown a cis effect, because the observed association could simply be a chance correlation (even after multiple-hypothesis correction). From the perspective of protein phosphorylation, the two ways that a SNP can act in cis are by changing the abundance of the molecule (data that the authors already have in hand) or by changing the coding sequence to alter docking sequences, localization, etc. This could be tested by sequencing the coding region of the proteins of interest in the samples (or, in at least a handful of samples). Otherwise, the entire basis of the cis finding is phenomenological.

Association studies identify alleles that occur more frequently in individuals with the higher and those with lower levels of kinase phosphorylation. We do not claim that association results identify the causative alleles. Mostly likely the most significant SNPs are in linkage disequilibrium with the causative variants. To clarify the point raised by the reviewer, we added the following in main text (Page 9):
"Further study is needed in order to identify functional variants that directly affect phosphorylation levels of target proteins."

Minor points
1. The addition of IGF1 stimulation as a positive control in Fig. S1A is welcome, but that positive control is so important that this experiment should replace the IP in Fig. 1A.
We incorporated Fig S1A into Fig.1A.

2. The total ERK and phospho-AKT blots on Fig. S3A look burned in, which will obscure possible quantitative differences among these lanes (PMID: 25852189). A lighter exposure should be shown.
The figures we show are with the lightest exposure. We have submitted source data of these figures.

3. The rightmost y-axis labels on Fig. S2 is cut off.
We have fixed it. We thank the reviewer for pointing it out.

4. Fig. 7A is missing a color bar to depict the range of values.
We have added the color bar. We thank the reviewer for pointing it out.

5. I missed this in the first review, but the expression of TIGAR and PER2 are not highlighted in Fig. 7A for high correlation with insulin-induced ERK phosphorylation. The authors should explain why the expression of TIGAR and PER2 are selected to validate the ERK-dependent gene expression in Fig. 7B.
In Fig. 7A, we highlighted the example genes on top of the panel and due to limited space we could not include TIGER and PER2. In Fig 7B, we include these two genes as additional examples, whose functions are interesting to us.

6. Gene symbols should be consistent in the manuscript. For example, the authors use TIGAR in Fig 7B, but C12orf5 in Supplementary Table S1.
We reviewed the gene symbols throughout the manuscript and made them consistent. The Supplementary Table S1 has been moved to GEO database per editor's request. We thank the reviewer for pointing it out.

7. Page 3 should read "food intake".
We have corrected it on Page 3. We thank the reviewer for pointing it out.

3rd Editorial Decision 16 June 2015

Thank you again for submitting your work to Molecular Systems Biology. We have now heard back from reviewer #2 who was asked to evaluate your study. As you will see below, this referee has two remaining concerns. Regarding the concern on Figure S3A, while we would welcome the inclusion of new blots if already available, we think that it is not mandatory.

Thank you for submitting this paper to Molecular Systems Biology.

Reviewer #2:

The authors have addressed most of the comments I had previously raised. The revised part regarding cis effect is much clear now. However, I still have two concerns and hope the authors could address them in the revision.
First, I suggest that the authors re-plot Fig. S3B. Fig. S3B shows the comparison between total levels of different proteins (left panel) and the comparison between insulin-stimulated phosphorylation of different proteins (right panel). It does not show the relationship between total level and induced phosphorylation of the same protein. As suggested in previous review, authors can plot fold-changes in phosphorylation vs. unstimulated Luminex fluorescence for the corresponding total protein. This is a clear way to support authors' point that unstimulated levels of total protein and insulin-induced phosphorylation are not correlated.
Second, the signals of total ERK and phospho-AKT on blots on Fig. S3A are saturated. This causes loss of quantitative accuracy. The author can avoid signal saturation by reducing the amounts loading protein or antibodies.

2nd Revision - authors' response 18 June 2015

We thank the reviewers for their comments and suggestions in this and previous reviews. We appreciate their help in improving our manuscript.

Reviewer #1:

The authors addressed most of my concerns, although it should still be stated a little more bluntly in the text what the physiological range of insulin concentrations are compared to what had to be used in vitro with the foreskin fibroblasts.

We follow the reviewer's suggestion and explicitly stated in the text the physiological insulin levels in human plasma (0.1~2 nM) (Page 5). The text reads:
"Human physiological plasma insulin levels are about 0.1~2 nM (Melmed et al, 2011); however, we expected a different concentration of insulin would be needed in cell-based experiments. To determine the optimal dose of insulin, we treated cells with serial titration of insulin and chose the concentration in the midpoint of dose-response curve (100nM), which is the same dose used for cultured fibroblast in other studies (Frittitta et al, 1998; Borisov et al, 2009)."

Also, the hits that were focused on, ENC1 and GEM, should be discussed in more detail. Maybe some info about what they do and how come they might be involved in the dynamic response to insulin across independent biological samples?

We discussed in detail the functions of ENC1 and GEM, and the implications of our findings (Page 11). The text reads:
"ENC1 encodes an actin-binding protein which is involved in adipocyte differentiation (Zhao et al, 2000). Its overexpression is also associated with various cancers (Fujita et al, 2001; Hammarsund et al, 2004; Durand et al, 2011). The positive correlation between ENC1 expression and ERK phosphorylation in response to insulin suggests a possible mechanism for higher cancer risk in patients with insulin resistance (Arcidiacono et al, 2012). GEM encodes an RGK family of GTPase, which regulates voltage-dependent Ca2+ channels. It has been shown that Gem-null mice were glucose intolerant and have impaired insulin secretion (Gunton et al, 2012). Here we showed that gene expression of GEM is regulated by insulin-stimulated ERK phosphorylation, suggesting a feedback pathway that between insulin secretion and insulin signaling."

Reviewer #2:

The authors have improved their manuscript in various aspects since the first submission. However two core issues, raised in the first review, still have not been satisfactorily addressed.

Major points
1. The authors should be commended for supplementing their phospho data with data on total protein. However, the display shown on Fig. S3B is not the comparison of interest. Clearly, the fold change of total proteins is not expected to change greatly upon acute insulin stimulation. The key comparison is whether the unstimulated levels of total protein ACROSS SAMPLES correlates with the extent of insulin stimulated phosphorylation. For example, one could envision a scenario where the ERK pathway is always stoichiometrically phosphorylated upon insulin stimulation; thus, the variation in observed ERK phosphorylation could be completely explained by the sample-to-sample variation in total ERK protein. The authors can readily assess this with their existing data by plotting fold-changes in phosphorylation vs. unstimulated Luminex fluorescence for the corresponding total protein.
In Fig S3B, we compare unstimulated total protein levels (left panel) and insulin-stimulated phosphorylation of same proteins (right panel) across sample. We showed that these two are not correlated, and therefore the variation exists mostly at the insulin-stimulated phosphorylation level, which cannot be explained by total protein level.

2. The authors make a big point to say that SNPs give rise to cis changes in phosphorylation. However, as multiple reviewers pointed out in the first review, the polymorphisms shown are all intronic, and therefore they are unlikely to be the causal variant. Therefore, the authors have not definitely shown a cis effect, because the observed association could simply be a chance correlation (even after multiple-hypothesis correction). From the perspective of protein phosphorylation, the two ways that a SNP can act in cis are by changing the abundance of the molecule (data that the authors already have in hand) or by changing the coding sequence to alter docking sequences, localization, etc. This could be tested by sequencing the coding region of the proteins of interest in the samples (or, in at least a handful of samples). Otherwise, the entire basis of the cis finding is phenomenological.

Association studies identify alleles that occur more frequently in individuals with the higher and those with lower levels of kinase phosphorylation. We do not claim that association results identify the causative alleles. Mostly likely the most significant SNPs are in linkage disequilibrium with the causative variants. To clarify the point raised by the reviewer, we added the following in main text (Page 9):
"Further study is needed in order to identify functional variants that directly affect phosphorylation levels of target proteins."

Minor points
1. The addition of IGF1 stimulation as a positive control in Fig. S1A is welcome, but that positive control is so important that this experiment should replace the IP in Fig. 1A.
We incorporated Fig S1A into Fig.1A.

2. The total ERK and phospho-AKT blots on Fig. S3A look burned in, which will obscure possible quantitative differences among these lanes (PMID: 25852189). A lighter exposure should be shown.
The figures we show are with the lightest exposure. We have submitted source data of these figures.

3. The rightmost y-axis labels on Fig. S2 is cut off.
We have fixed it. We thank the reviewer for pointing it out.

4. Fig. 7A is missing a color bar to depict the range of values.
We have added the color bar. We thank the reviewer for pointing it out.

5. I missed this in the first review, but the expression of TIGAR and PER2 are not highlighted in Fig. 7A for high correlation with insulin-induced ERK phosphorylation. The authors should explain why the expression of TIGAR and PER2 are selected to validate the ERK-dependent gene expression in Fig. 7B.
In Fig. 7A, we highlighted the example genes on top of the panel and due to limited space we could not include TIGER and PER2. In Fig 7B, we include these two genes as additional examples, whose functions are interesting to us.

6. Gene symbols should be consistent in the manuscript. For example, the authors use TIGAR in Fig 7B, but C12orf5 in Supplementary Table S1.
We reviewed the gene symbols throughout the manuscript and made them consistent. The Supplementary Table S1 has been moved to GEO database per editor's request. We thank the reviewer for pointing it out.

7. Page 3 should read "food intake".
We have corrected it on Page 3. We thank the reviewer for pointing it out.

© European Molecular Biology Organization
