# Supplementary material for: Genetic variation in insulin-induced kinase signaling
Source: Mol Syst Biol. 2015 Jul 22;11(7):820. doi: 10.15252/msb.20156250 (PMC4547848; doi:10.15252/msb.20156250)
Supplement: Supplementary file 5 [file msb0011-0820-sd5.zip › SourceDataFig1/Source Data for Figure 1A/Fig1A_source data annotated.pdf]

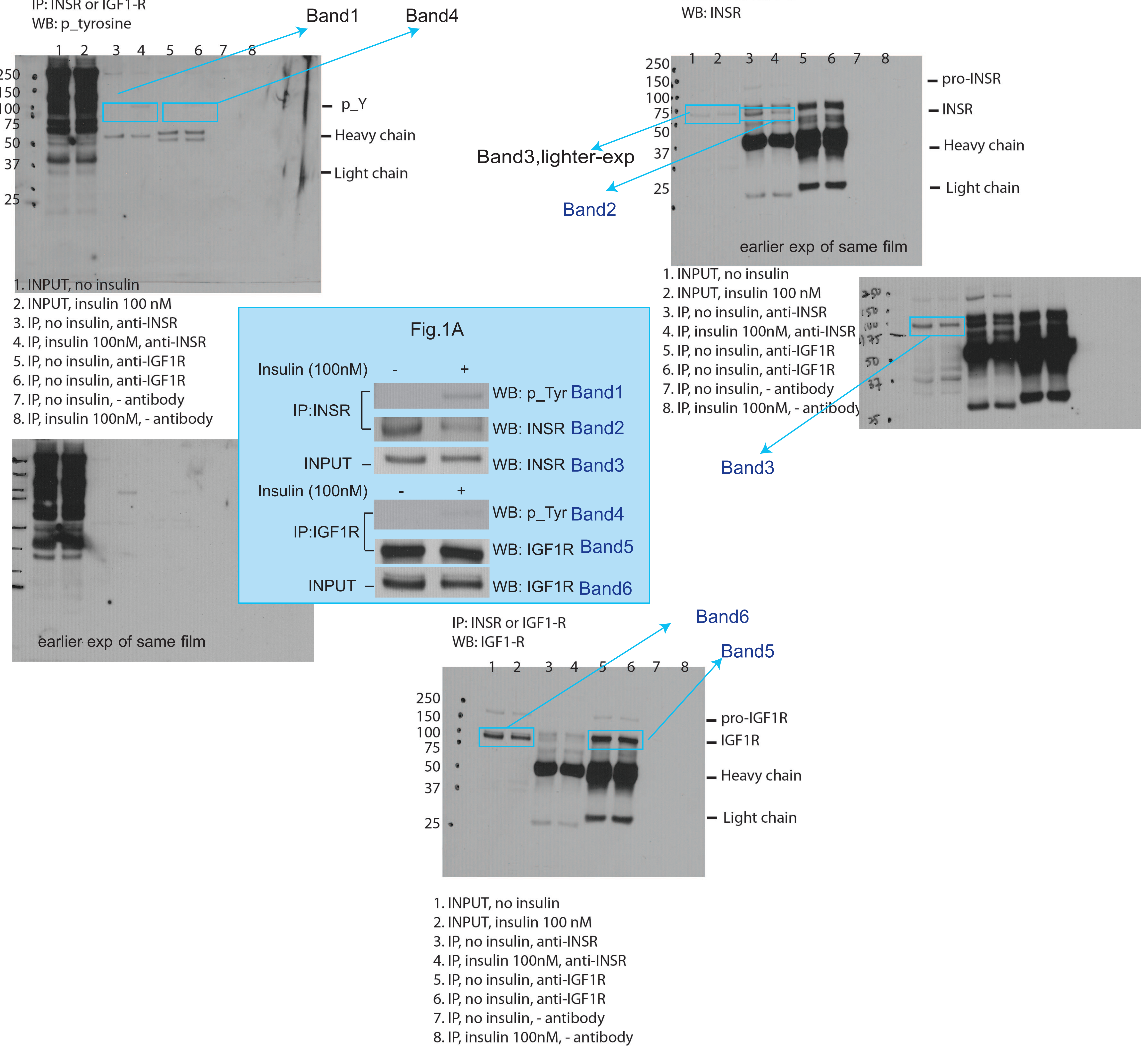
IP: INSR or IGF1-R
WB: p_tyrosine
Band1
Band4
1 2 3 4 5 6 7 8
250
150
100
75
50
37
25
p_Y
Heavy chain
Light chain
1. INPUT, no insulin
2. INPUT, insulin 100 nM
3. IP, no insulin, anti-INSR
4. IP, insulin 100nM, anti-INSR
5. IP, no insulin, anti-IGF1R
6. IP, no insulin, anti-IGF1R
7. IP, no insulin, - antibody
8. IP, insulin 100nM, - antibody
earlier exp of same film
WB: INSR
1 2 3 4 5 6 7 8
250
150
100
75
50
37
25
pro-INSR
INSR
Heavy chain
Light chain
Band3,lighter-exp
Band2
earlier exp of same film
1. INPUT, no insulin
2. INPUT, insulin 100 nM
3. IP, no insulin, anti-INSR
4. IP, insulin 100nM, anti-INSR
5. IP, no insulin, anti-IGF1R
6. IP, no insulin, anti-IGF1R
7. IP, no insulin, - antibody
8. IP, insulin 100nM, - antibody
Band3
Fig.1A
Insulin (100nM) - +
IP:INSR
WB: p_Tyr Band1
WB: INSR Band2
INPUT
WB: INSR Band3
Insulin (100nM) - +
IP:IGF1R
WB: p_Tyr Band4
WB: IGF1R Band5
INPUT
WB: IGF1R Band6
IP: INSR or IGF1-R
WB: IGF1-R
Band6
Band5
1 2 3 4 5 6 7 8
250
150
100
75
50
37
25
pro-IGF1R
IGF1R
Heavy chain
Light chain
1. INPUT, no insulin
2. INPUT, insulin 100 nM
3. IP, no insulin, anti-INSR
4. IP, insulin 100nM, anti-INSR
5. IP, no insulin, anti-IGF1R
6. IP, no insulin, anti-IGF1R
7. IP, no insulin, - antibody
8. IP, insulin 100nM, - antibody
