# Supplementary figures and images for: Genetic variation in insulin-induced kinase signaling
Source: Mol Syst Biol. 2015 Jul 22;11(7):820. doi: 10.15252/msb.20156250 (PMC4547848; doi:10.15252/msb.20156250)

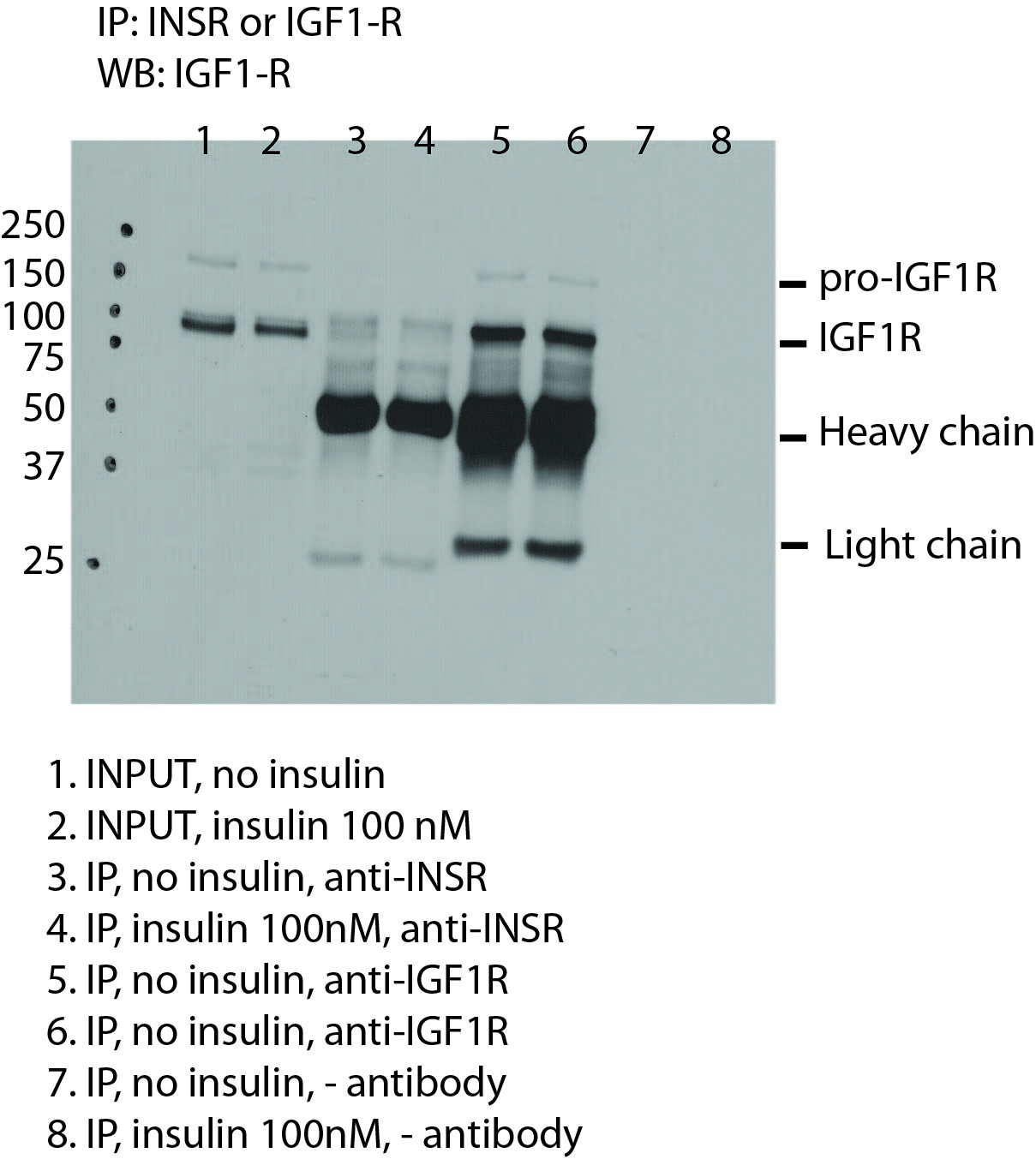

Supplement: Supplementary file 5 [file msb0011-0820-sd5.zip › SourceDataFig1/Source Data for Figure 1A/1A_left IGF1R.jpg]

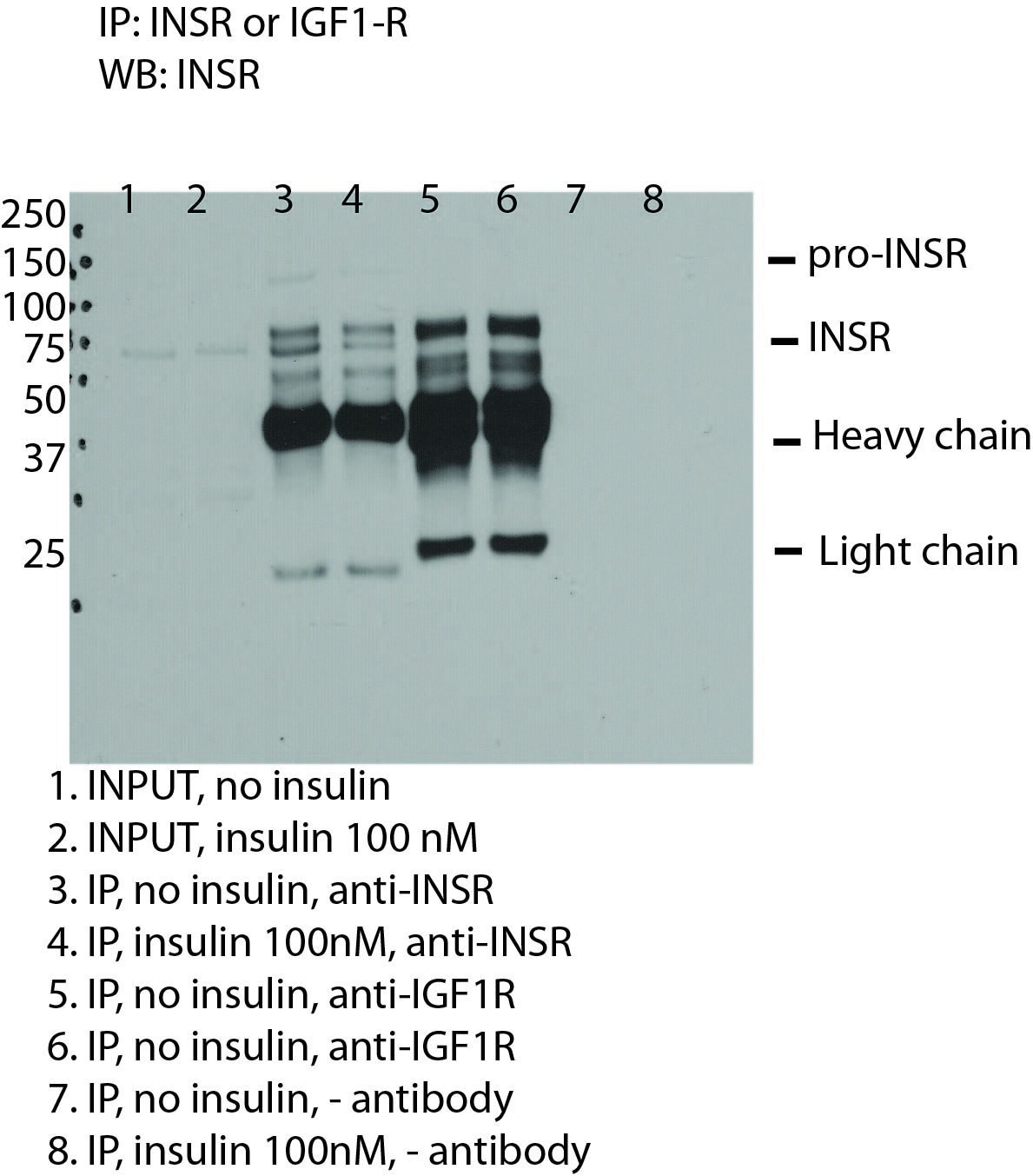

Supplement: Supplementary file 5 [file msb0011-0820-sd5.zip › SourceDataFig1/Source Data for Figure 1A/1A_left INSR_short exposure.jpg]

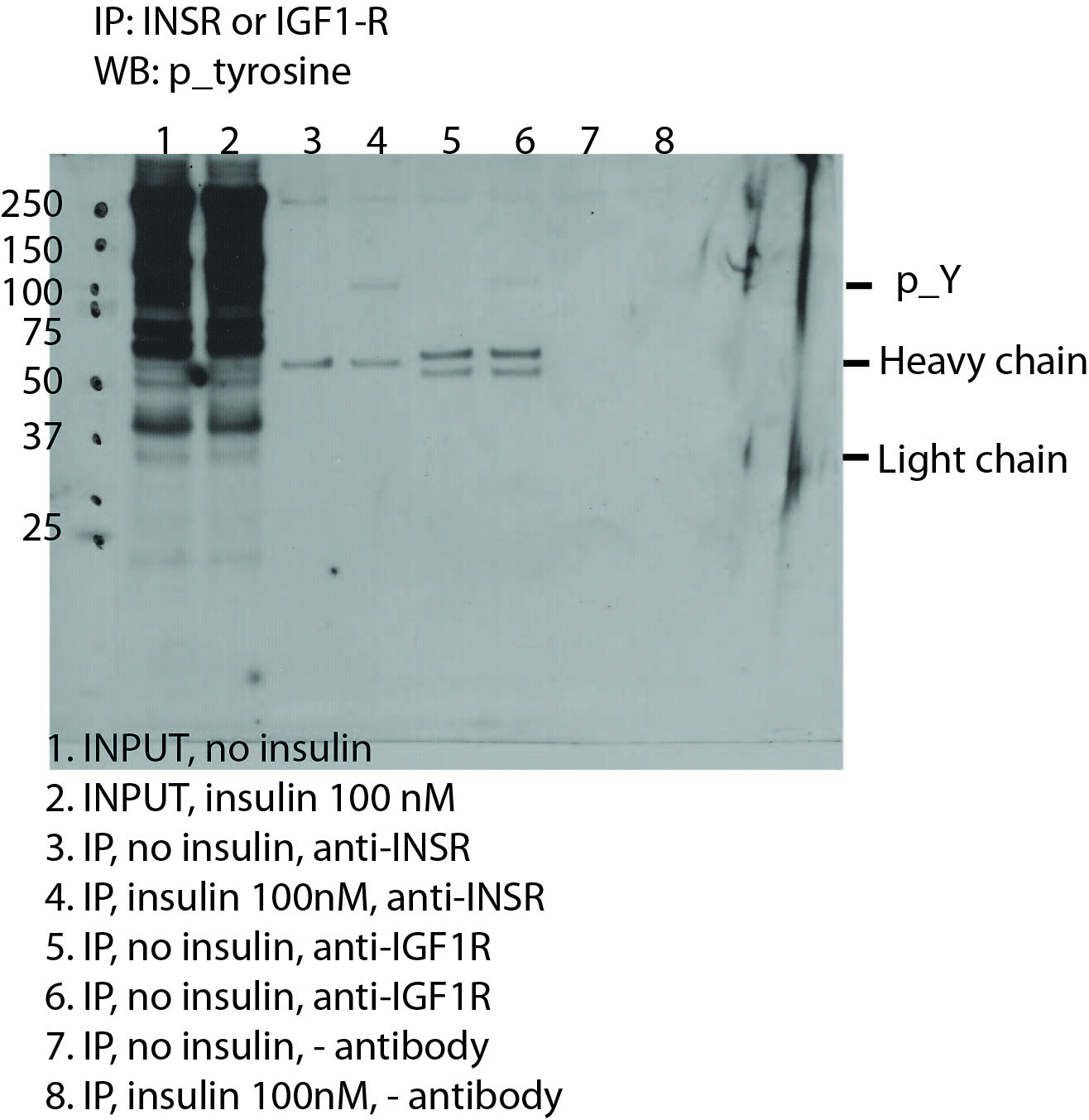

Supplement: Supplementary file 5 [file msb0011-0820-sd5.zip › SourceDataFig1/Source Data for Figure 1A/1A_left pY longer exposure.jpg]

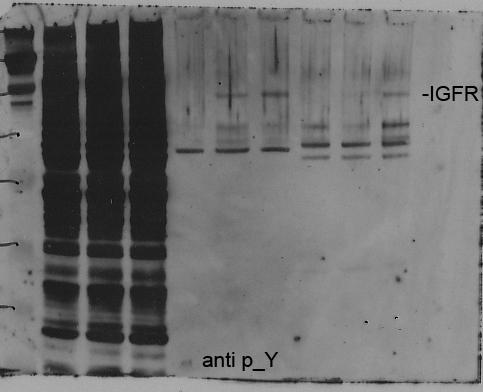

Supplement: Supplementary file 5 [file msb0011-0820-sd5.zip › SourceDataFig1/Source Data for Figure 1A/1A_right pY.tif]

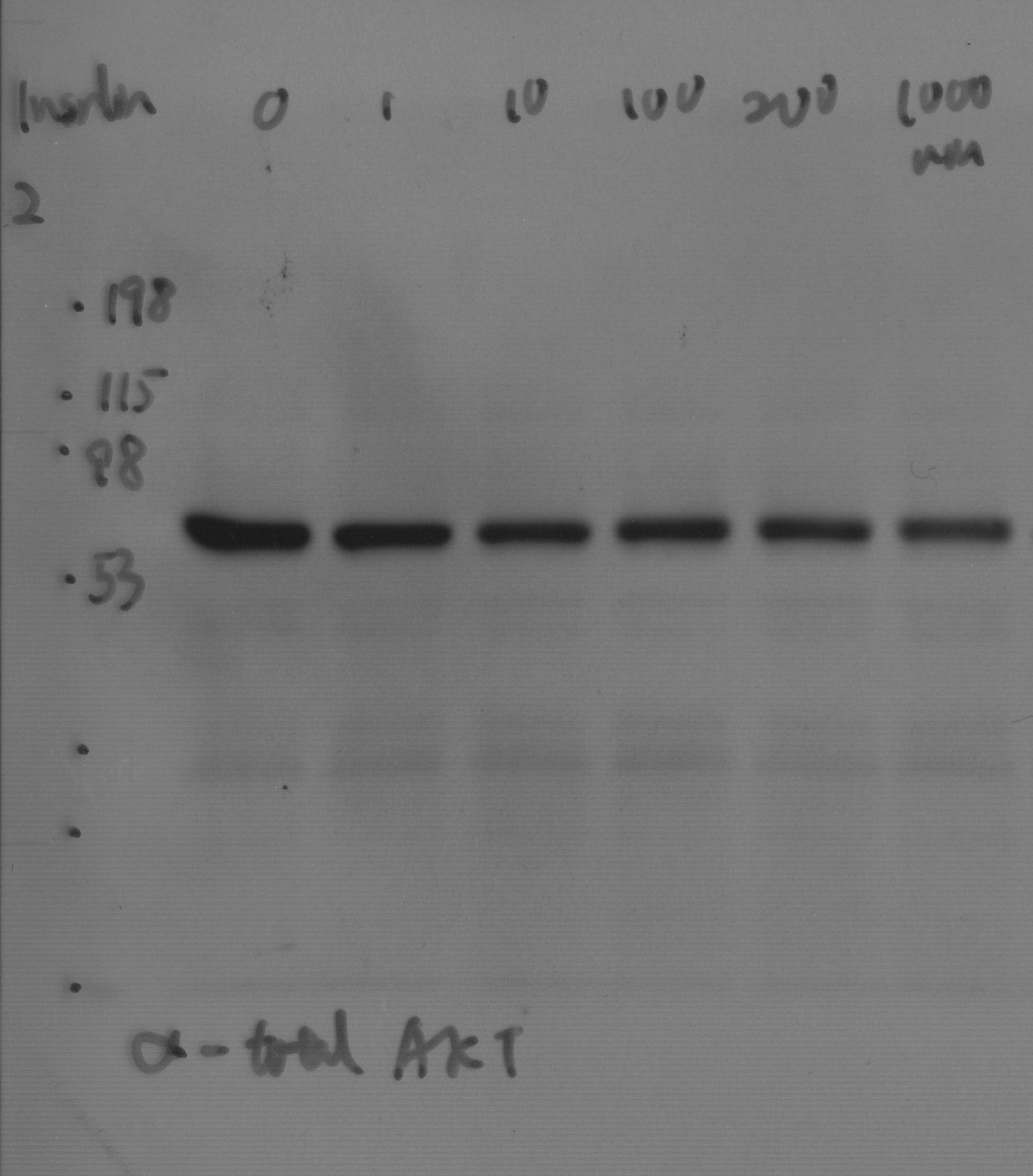

Supplement: Supplementary file 5 [file msb0011-0820-sd5.zip › SourceDataFig1/Source Data for Figure 1B/1B AKT.tif]

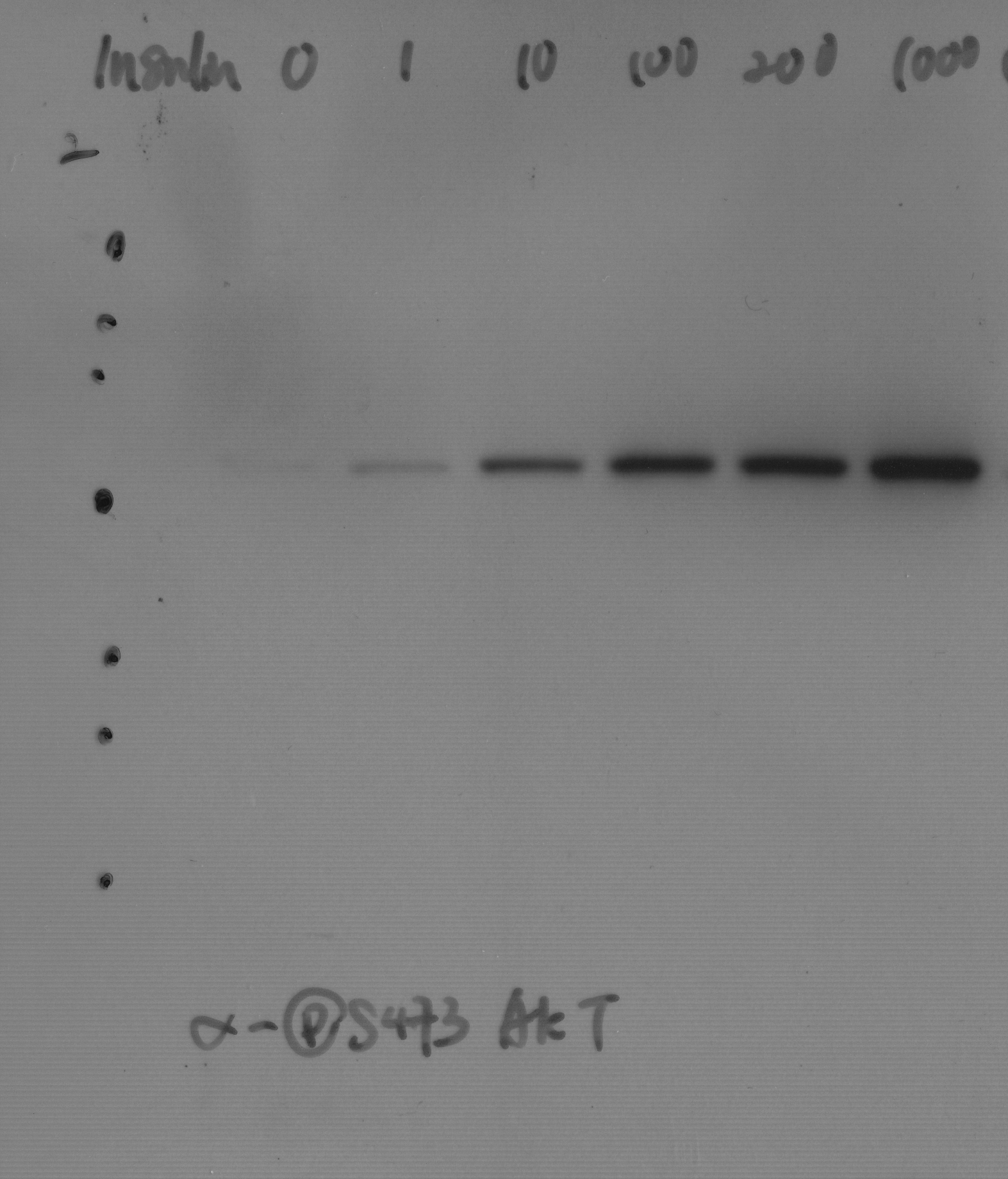

Supplement: Supplementary file 5 [file msb0011-0820-sd5.zip › SourceDataFig1/Source Data for Figure 1B/1B pAKT.tif]

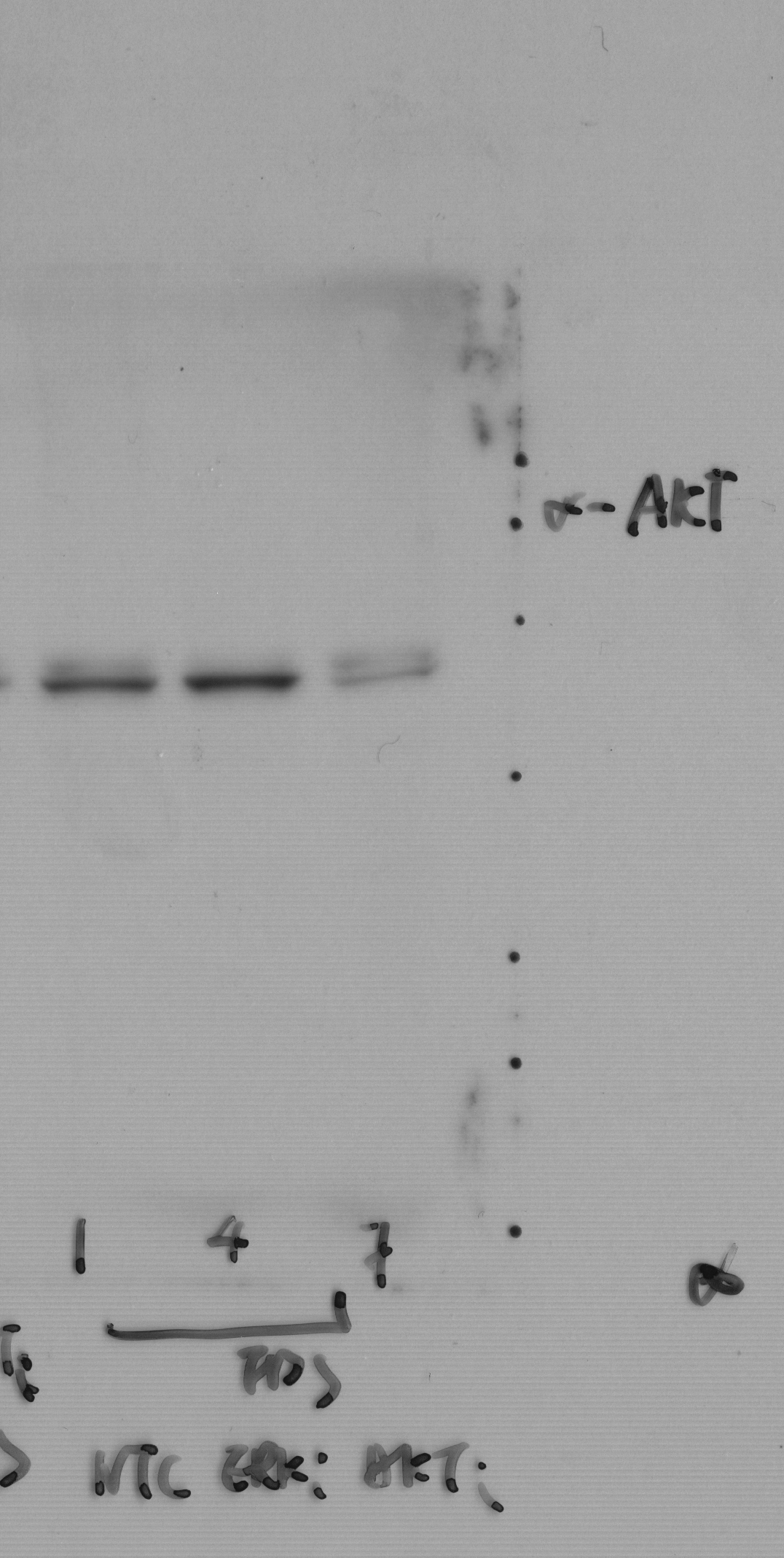

Supplement: Supplementary file 8 [file msb0011-0820-sd8.zip › Source Data for Figure 7E_test/Source Data for Figure 7E/7E AKT.tif]

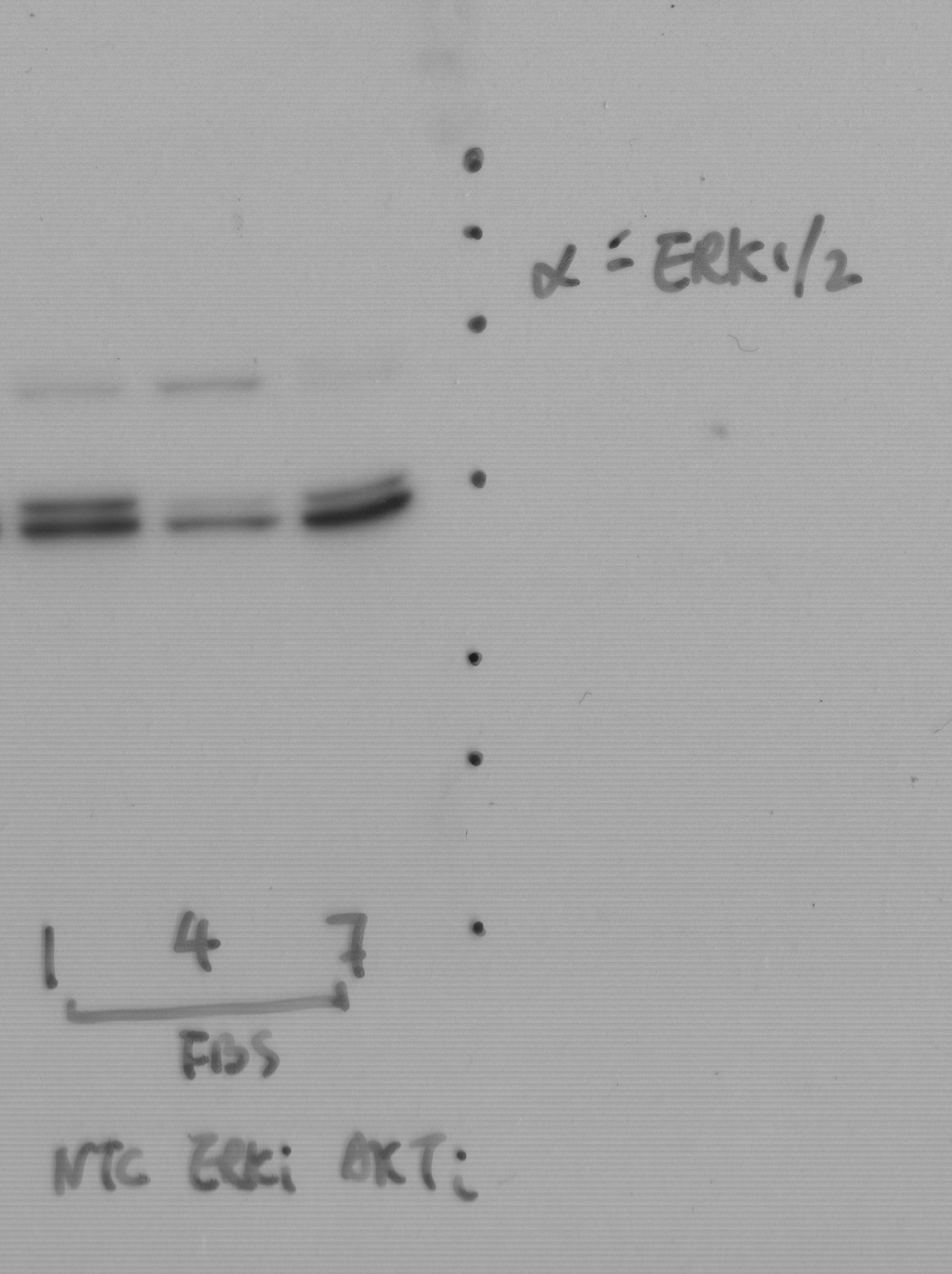

Supplement: Supplementary file 8 [file msb0011-0820-sd8.zip › Source Data for Figure 7E_test/Source Data for Figure 7E/7E ERK1_2.tif]

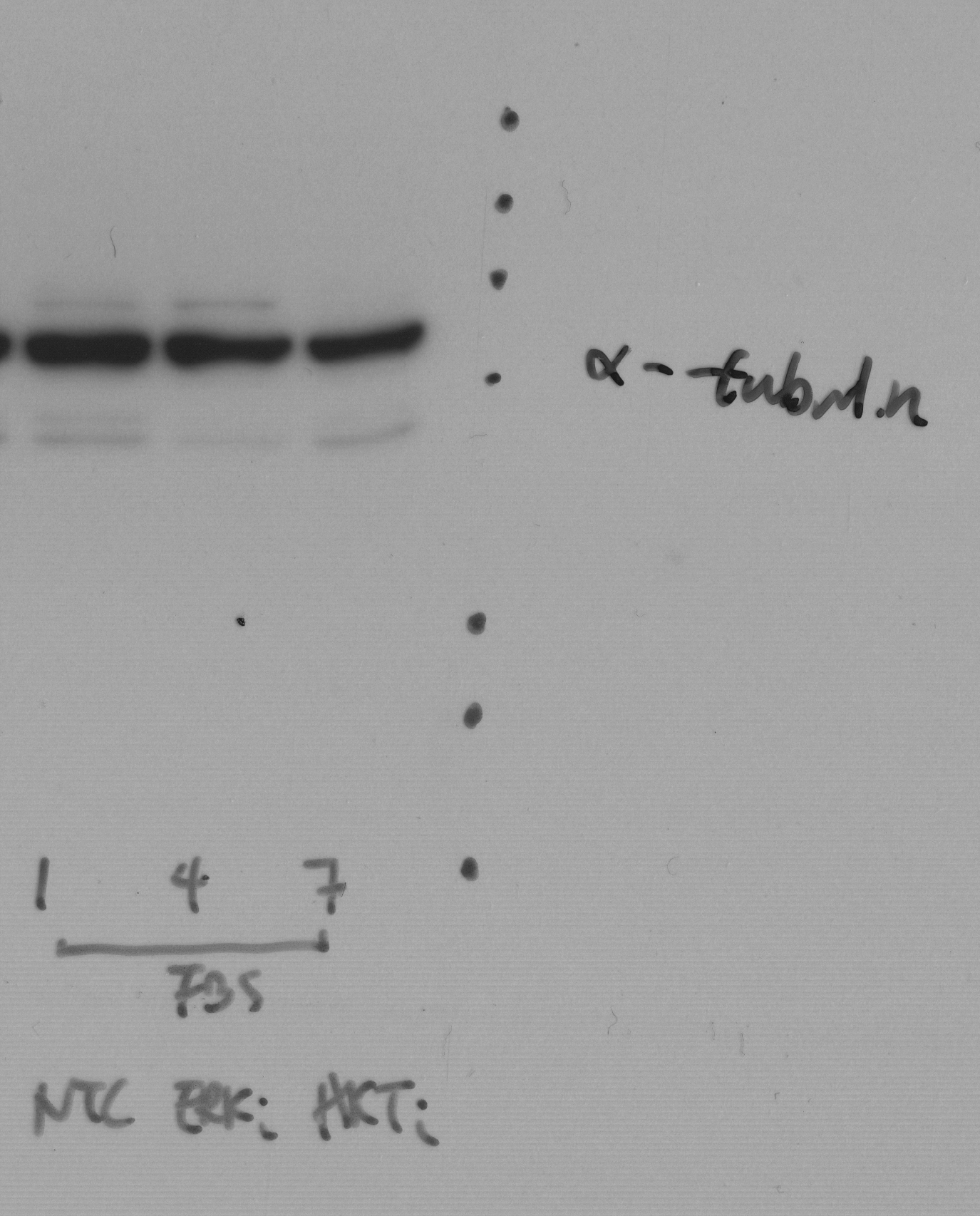

Supplement: Supplementary file 8 [file msb0011-0820-sd8.zip › Source Data for Figure 7E_test/Source Data for Figure 7E/7E tubulin.tif]

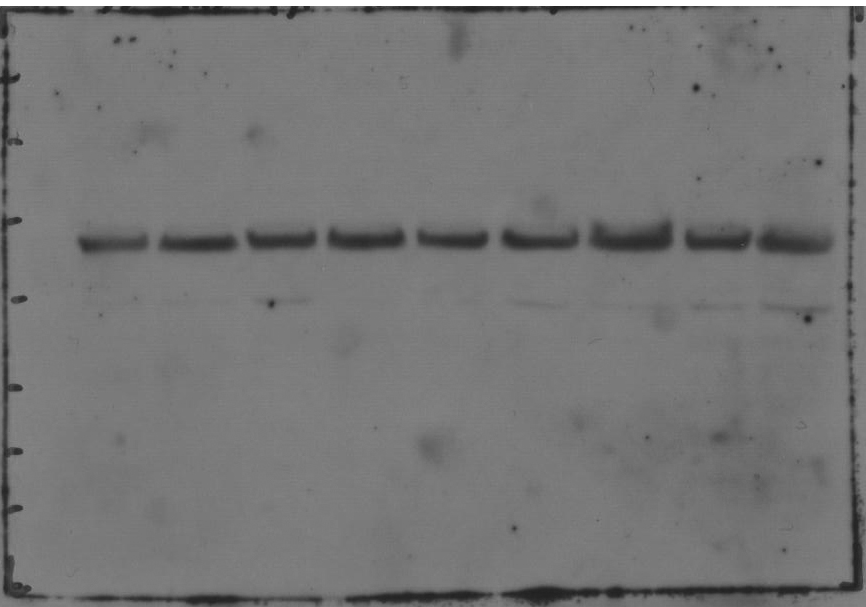

Supplement: Supplementary file 9 [file msb0011-0820-sd9.zip › Source Data Expanded View Figures/Source Data_FigEV3_AKT.tif]
